# Boosting prairie dog optimizer for optimal planning of multiple wind turbine and photovoltaic distributed generators in distribution networks considering different dynamic load models

**DOI:** 10.1038/s41598-024-64667-4

**Published:** 2024-06-19

**Authors:** Mohamed A. Elseify, Fatma A. Hashim, Abdelazim G. Hussien, Hussein Abdel-Mawgoud, Salah Kamel

**Affiliations:** 1 Department of Electrical Engineering, Faculty of Engineering, Al-Azhar University, Qena, 83513 Egypt; 2 Faculty of Engineering, Helwan University, Cairo, Egypt; 3 Faculty of Information Technology, Middle East University, Amman, 11831 Jordan; 4 Department of Computer and Information Science, Linköping University, 581 83 Linköping, Sweden; 5 Faculty of Science, Fayoum University, Fayoum, 63514 Egypt; 6 Department of Electrical Engineering, Faculty of Engineering, Aswan University, Aswan, 81542 Egypt

**Keywords:** Uncertainty, DG optimal planning, Distribution system, PDO algorithm, Energy loss, Photovoltaic, Wind turbine, Slim mould algorithm, Time variant load, Applied mathematics, Computer science, Software, Electrical and electronic engineering

## Abstract

Deploying distributed generators (DGs) supplied by renewable energy resources poses a significant challenge for efficient power grid operation. The proper sizing and placement of DGs, specifically photovoltaics (PVs) and wind turbines (WTs), remain crucial due to the uncertain characteristics of renewable energy. To overcome these challenges, this study explores an enhanced version of a meta-heuristic technique called the prairie dog optimizer (PDO). The modified prairie dogs optimizer (mPDO) incorporates a novel exploration phase inspired by the slime mold algorithm (SMA) food approach. The mPDO algorithm is proposed to analyze the substantial effects of different dynamic load characteristics on the performance of the distribution networks and the designing of the PV-based and WT-based DGs. The optimization problem incorporates various operational constraints to mitigate energy loss in the distribution networks. Further, the study addresses uncertainties related to the random characteristics of PV and WT power outputs by employing appropriate probability distributions. The mPDO algorithm is evaluated using cec2020 benchmark suit test functions and rigorous statistical analysis to mathematically measure its success rate and efficacy while considering different type of optimization problems. The developed mPDO algorithm is applied to incorporate both PV and WT units, individually and simultaneously, into the IEEE 69-bus distribution network. This is achieved considering residential, commercial, industrial, and mixed time-varying voltage-dependent load demands. The efficacy of the modified algorithm is demonstrated using the standard benchmark functions, and a comparative analysis is conducted with the original PDO and other well-known algorithms, utilizing various statistical metrics. The numerical findings emphasize the significant influence of load type and time-varying generation in DG planning. Moreover, the mPDO algorithm beats the alternatives and improves distributed generators' technical advantages across all examined scenarios.

## Introduction

Renewable distributed generators integrated into distribution networks have multiple benefits in terms of technological, economic, and environmental aspects. It can be classified into four types according to whether electrical power is injected into the grid or absorbed from the network^1^. Wind and solar energy technologies are the most prevalent among these categories. Wind turbine (WT) technology and geographical location are two factors that affect the penetration of wind energy-based DG into the electric utility. Nevertheless, greater degrees of penetration might result in adverse effects such as heightened voltage fluctuations and power losses in the network. On the other hand, the amount of solar radiation that a photovoltaic (PV) system receives at the installation site largely determines the power it produces. However, by effectively integrating WT and PV systems, it is possible to achieve a degree of penetration that guarantees the smooth functioning of the electrical grid.

Distributed generators (DGs) are typically installed on the distribution side of the electrical utility due to the higher ratio of R/X compared to the transmission side. A high R/X ratio indicates increased power loss across distribution networks^2^. Therefore, integrating DGs into distribution networks (DNs) plays a vital role in minimizing line losses of the systems, enhancing system reliability, refining voltage stability, maintaining system integrity, and improving voltage profiles. However, improper placement of DG units can undermine these important characteristics. Moreover, the intermittent nature of solar radiation and wind speed in PV systems and WTs poses challenges for their optimal allocation in DNs in terms of technical, financial, and environmental aspects^3,4^. The literature has extensively examined the implications of disregarding the uncertainties in demand and generation during the installation of renewable energy sources^1,5,6^. However, these studies have not addressed realistic scenarios involving time-varying load and generation, as the capacity and location of DG may differ under varying loading levels, particularly at peak demand.

Several research studies have employed various analytical expressions^7–9^ and heuristic or meta-heuristic optimization algorithms to address numerous challenges in DG allocation issues. In Ref.^7^, the authors proposed an analytical formulation based on DG operating at unity power factor (UPF) to identify the optimal location using demand data, admittance matrix, and generation data. However, this approach is suitable only for a single DG unit. Another analytical technique, presented in Ref.^8^, utilized a multi-objective index considering time-varying load models to allocate a single DG unit based on active power loss, voltage deviation, and reactive power loss. Nevertheless, it is limited to DG installations with a UPF. Freshly, the authors of Ref.^9^ studied the impact of time-varying voltage-dependent load profiles on hybrid PV and WT systems using a multi-objective index. This index incorporates apparent power capacity, voltage fluctuation, and active/reactive loss, considering uncertainties in wind speed and solar irradiance to improve voltage profiles, mitigate power loss, and reduce line capacity limits. However, it also focused on a single PV and WT unit with consideration of different load profiles. In addition to analytical approaches, multiple meta-heuristic algorithms have been proposed to address the DG optimal allocation challenge^10,11^. However, they overlook the probabilistic behavior of renewable energy sources and assume numerous voltage-dependent load models. For illustration, the authors of Ref.^10^ proposed a framework based on the multi-objective Lichtenberg and thermal exchange optimization techniques for installing three units of capacitor banks and DGs with different operating power factors, considering eight different load models, to enhance voltage stability while alleviating power loss and voltage deviation. In Ref.^11^, the weighted multi-objective stud Krill herd algorithm has been utilized to optimize the allocation of multiple PV-based DGs under varied load models, aiming to maximize the technical benefits of the PV generation system. In Ref.^12^, the hybrid gray wolf and PSO algorithms are suggested to address the optimal inclusion of multiple DGs in a realistic Dilla distribution system. The goal is to enhance the voltage level and lower both active and reactive power losses. The aggregated multi-objective coefficient approach is used to combine these various objectives into a single objective.

Efficient optimization algorithms have been employed to specify the optimal sizing and capacity of a single DG unit under time-variant voltage-dependent load profiles in several studies^13–16^. As an illustration, in Ref.^13^, a hybrid renewable DG system combining PV, WT, and battery energy units was proposed using an improved version of the Crow search algorithm to decrease energy loss and voltage fluctuations under varying load levels. Monte Carlo simulation was utilized to model the uncertainties associated with PV and WT systems. In Ref.^14^, a novel Weibull function-based time-coupled probabilistic generation model was developed to determine a suitable location and capacity of WT. The particle swarm optimization algorithm was used to address the multi-objective optimization model, which aims at minimizing reactive and actual losses, maximizing voltage stability, and mitigating voltage drop. The authors of Ref.^15^ introduced a hybrid technique combining slap swarm and PSO algorithms to specify the optimum sitting and sizing of a single WT and PV unit, integrated individually or simultaneously into distribution systems, with the aim of minimizing technical objectives related to the WT unit. In Ref.^16^, the slap swarm optimizer was utilized to optimally assign a single WT unit on 33 and 69-bus DNs. The study further investigated the influence of various time-variant voltage-dependent load demands, including constant, commercial, industrial, residential, and mixed types, on distribution system performance and optimal DG planning problems.

Furthermore, the optimal integration of multiple WT and PV units into different radial DNs was addressed in Ref.^17^ using the slap swarm optimization technique. However, this approach did not simulate the stochastic nature of load and generating units. Authors of Refs.^18,19^ presented approaches for determining the optimal deployment of PV and WT units in small-scale DNs, considering the stochastic behavior of load and generation, to reduce annual energy loss. In Ref.^20^, the simulated annealing algorithm was combined with the manta ray foraging method to enhance the exploitation rate of the random foraging operator, which was used for optimal sizing of an inverter-based PV unit integrated into 69-bus test system under commercial load type. In this study, the battery energy storage (BES) was incorporated with the non-dispatchable PV to transform it into a dispatchable source. However, the convergence characteristics of these algorithms were not discussed. In Ref.^21^, the integration of PV with BES system into 33-bus and 69-bus DNs have been accomplished using the non-dominated multi-objective manta ray foraging optimizer algorithm, presuming plug-in electric vehicles. The multi-objective seeks to capture maximum techno-economic benefits of the PV-BES units. In Ref.^22^, the integration of single and double WT and PV units, with UPF and optimal power factor (OPF), into a 33-bus DN was proposed using two heuristic algorithms, namely genetic algorithm and PSO, with the aim of reducing aggregated multi-objectives of annual energy losses and voltage deviation index. In this study, the uncertainties associated with generation and load across four seasons have been investigated. In Ref.^23^, the archimedes optimizer (AO) was utilized to find the appropriate location and capacity of single and multiple PVs integrated simultaneously with battery energy storage systems under commercial, residential, and industrial load profiles. The main goal of the AO was to mitigate active power loss in the 69-bus DN for one practical day, taking into account equality and inequality restrictions. Nonetheless, the efficacy and superiority of the proposed approach have not been measured.

However, several modification theories are incorporated into conventional optimization techniques to strengthen their convergence characteristics and boost the exploitation and exploration stages from diverse perspectives. In other words, using advanced optimization methods results in obtaining multiple benefits and achieving superior results. In this context, the authors of Ref.^24^ boosted the artificial ecosystem-based optimizer to identify the appropriate placement and sizing of WT and PV units, considering the unpredictable behavior of solar irradiance and wind speed, with the goal of minimizing energy loss in 33-bus and 85-bus DNs. The developed algorithm incorporated five powerful methods: improved linear weight coefficient, production operator, enhanced consuming operator, enhanced decomposition operator, and opposition-based learning. In Ref.^25^, a hybrid technique integrating PSO and gravitational search algorithms was developed to optimize the economic and technological advantages of WT and PV systems while accounting for seasonal fluctuations in demand and generation. A study reported in Ref.^26^ hybridized the simulated annealing algorithm with the Henry gas solubility optimizer to enhance the exploitation rate of the original HGS. The proposed hybrid HGS algorithm was utilized for strategic planning of PV and BES hybrid units to lessen daily active power loss in the 69-bus system under commercial load demand. However, the numerical findings showed the slow convergence characteristics of the developed algorithm. The authors of Ref.^27^ enhanced the sunflower algorithm to boost the performance of distribution networks by optimizing both wind turbine placement and network reconfiguration. A Monte Carlo simulator was used to replicate the intermittent nature of wind speed, while two load profiles were used to simulate the variation in demand over a year. The enhanced algorithm incorporated an adaptive pollination rate and an adaptive sunflower inertial displacement to improve its convergence characteristics and solution quality. Ultimately, the authors of Ref.^28^ applied the basic artificial hummingbird optimizer for allocating single PV and WT synchronously in the 69-bus DN with consideration of the intermittences associated with solar radiation and wind speed in WT and PV systems. The studied multi-objective function is optimized to raise the techno-economic benefits of the DGs.

Previous research has only conducted a few investigations on the fluctuations in demand and electricity generated by PV and WT renewable DG sources. In other words, the most common load profile studied in the literature was the commercial load type. In conclusion, the performance of DNs and the challenges associated with DG optimal planning are influenced by the type of load^9,14–16^. Additionally, the uncertainties in generation and load further complicate the DG optimal planning problem. It is well-established that improving the performance of optimization algorithms leads to significant benefits and more optimal solutions, as discussed in Refs.^26,27,29,30^. Therefore, this study focuses specifically on the effects of individually and concurrently assigning various WTs and PVs on the operation of the distribution system. The analysis takes into account the load profiles of commercial, residential, industrial, and mixed sectors. Further, the stochastic nature of the wind speed and solar irradiance is simulated using efficient probability distribution functions. The optimal DG allocation challenge is achieved using an enhanced variant of the prairie dog optimization (PDO) method^31^, which is a novel meta-heuristic technique that imitates the habits of prairie dogs in their natural environment.

The PDO incorporates four behaviors of prairie dogs to accomplish the exploration and exploitation phases, which are essential stages in optimization. The foraging activity and cave construction of prairie dogs are utilized to support the algorithm's exploratory behavior. When the food supply becomes exhausted, prairie dogs scour the whole colony and its environs for other food sources. Upon finding alternative sources of food, they quickly start building burrows in that area. Utilizing prairie dogs' distinctive responses to various warning or communication noises makes the exploitation phase simpler. They emit noises or alerts in various situations, such as the existence of predators or the presence of food. Communication plays a crucial role in meeting their nutritional requirements and ensuring their safety against potential assailants. In the case of the PDO algorithm, these two characteristics drive the prairie dogs to converge at a certain position or a desirable location, where further search (exploitation) is carried out to identify more favorable or nearly optimum solutions. PDO has recently been applied to address damage identification challenges in engineering structures^32^, which indicated the need for enhancing the algorithm to handle extensive optimization challenges.

Therefore, in this work, the slime mold algorithm (SMA) is employed to enhance the exploration capabilities of the standard PDO. The performance of the modified prairie dog optimizer (mPDO) is evaluated by different statistical analyses and compared with well-known competitors based on the standard cec2020 benchmark functions. Subsequently, the original and mPDO algorithms are applied to identify the optimal planning of single and multiple PVs and WTs incorporated into 69-bus DN, considering a snapshot of generation and load, while mitigating active losses of the system. The simulation outcomes are compared with those of known optimization approaches, including the new heuristic approach (NHA)^5^ and the novel stochastic fractal search algorithm (SFSA)^6^. Finally, the mPDO is applied to specify the optimal deployment of single and multiple PVs and WTs under various time-variant voltage-dependent loads, including commercial, residential, and industrial sectors. Further, several performance metrics are utilized to measure the efficacy of the present test system. The study also investigates the combined installation of PV and WT units in the presence of various load patterns. The findings proved the success rate of the mPDO in handling the optimal allocation challenge of DG when both generation and demand are unpredictable. The following is a summary of the key innovations and contributions of this study:

- Enhancing the performance of the novel prairie dog optimization algorithm by incorporating the reinforced learning slime mold algorithm, resulting in the modified mPDO algorithm. Further, the success and superiority of the mPDO are verified through its implementation on the cec2020 test suite benchmark and rigorous statistical analysis.
- Applying suitable probability distributions to model the ambiguity caused by solar irradiation and wind velocity, enabling the simulation of power variability in WT and PV units. Additionally, dynamic load characteristics representing industrial, commercial, residential, and mixed sectors are have been deemed to capture changes in demand throughout the day.
- Employing the mPDO to obtain the optimal inclusion of single and multiple WT and PV systems in 69-bus DN with the goal of lowering the daily active power loss, considering both deterministic and stochastic scenarios.
- Investigating the impacts of different dynamic load patterns on the performance of DN and the scheduling of the PV and WT units. This analysis provides insights into the optimal utilization of these renewable energy sources under varying load conditions.
- The numerical outcomes highlighted the robustness and reliability of the mPDO in addressing the optimal installation challenges of the PV and WT systems, particularly in the existence of uncertainty related to supply and demand.

The subsequent parts of this article are structured in the following manner. In the next section, the objective function, operating constraints, and mathematical expressions related to PV system modeling, WT modeling, and time-variant load modeling are presented. Section "Probabilistic models of generation and demand" introduces the proposed methodology and its implementation. The numerical solutions are extensively discussed in Section "Numerical simulation and discussions". Eventually, Sect. “Conclusion and future work” outlines a summary of the outcomes and offers suggestions for future research.

## Problem expression

### Objective function

This paper addresses the allocation problem of a non-dispatchable renewable DG, such as PV and WT systems, aiming to optimize the objective function, which involves reducing network energy loss under uncertainties related to generation and demand. The modified algorithm is employed to achieve this optimization, considering diverse load patterns such as residential, industrial, and commercial profiles. Though, it is crucial to ensure that the installation of DGs in the distribution network adheres to operational constraints to maintain system integrity.

**Figure 1:**
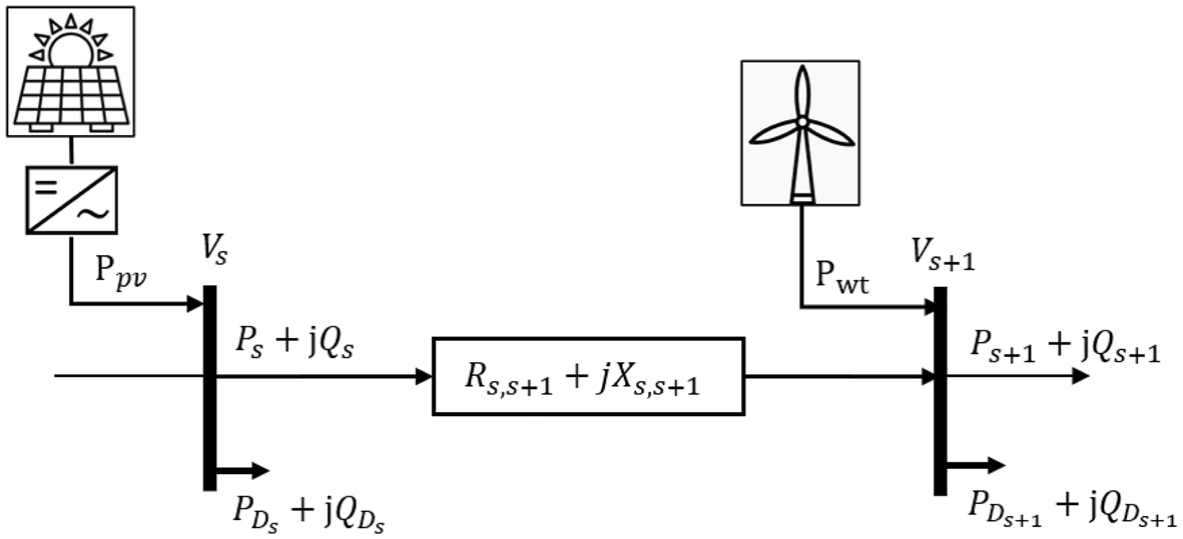
Part of distribution network with an integrating PV at bus $s$ and WT at bus $(s+1)$ simultaneously.

Let us consider a two-node network $(s, s+1)$ illustrated in Fig. 1 with $n$ nodes and $(n-1)$ lines. The connecting line has a resistance and reactance of ${(R}_{s,s+1}+j{X}_{s,s+1})$. In such systems, the supply is the substation, in which the real and reactive line flow between every two connecting buses are respectively represented by ${P}_{s}$ and ${Q}_{s}$. Let the reactive and active demand power are symbolized by ${Q}_{d}$ and ${P}_{d}$, respectively. In this context, after integrating a unit of WT into bus $(s+1)$ while supplying real and reactive power of ${P}_{wt,s+1}$ and ${Q}_{wt,s+1}$, respectively, the forward line flow equations of the backward-forward sweep (BFS) algorithm can be expressed as below^33^. The BFS is executed with a tolerance of 10^6^ to solve the distribution system load flow.

1 $${P}_{s+1}={P}_{wt,s+1}+{P}_{s}-{P}_{D,s+1}-{R}_{s,s+1}\left(\frac{{P}_{s}^{2}+{Q}_{s}^{2}}{{\left|{V}_{s}\right|}^{2}}\right) ,$$

2 $${Q}_{s+1}={Q}_{wt,s+1}+{Q}_{s}-{Q}_{D,s+1}-{X}_{s,s+1}\left(\frac{{P}_{s}^{2}+{Q}_{s}^{2}}{{\left|{V}_{s}\right|}^{2}}\right),$$

3 $${V}_{s+1}^{2}={V}_{s}^{2}-2\left({R}_{s,s+1} {P}_{s}+{X}_{s,s+1} {Q}_{s}\right)+\left({P}_{s}^{2}+{Q}_{s}^{2}\right)\left(\frac{{\left|{R}_{s,s+1}+j{X}_{s,s+1}\right|}^{2}}{{\left|{V}_{s}\right|}^{2}}\right),$$

Accordingly, the active line loss in a branch of a DN, i.e. $(s, s+1)$, can be estimated using the expression (4) as,

4 $${P}_{l,(s,s+1)}={R}_{s,s+1}\left(\frac{\left({{P}_{s+1}}^{2}+{{Q}_{s+1}}^{2}\right)}{{\left|{V}_{s}\right|}^{2}}\right),$$

The total active power $({P}_{LOSS})$ dissipation across the whale network, encompassing all branches, can be evaluated by summing up the loss across all lines of the DN, as given in Eq. (5).

5 $${P}_{LOSS}=\sum_{i=1}^{n-1}{P}_{l,i} ,$$

The main focus of optimal DG installation problems is to boost the positive effects while mitigating the negative impacts on power systems. Therefore, the key contribution of the paper is to develop the capabilities of the PDO method in determining the optimal locations for single and multiple DGs, with the objective of diminishing the daily active line loss as defined in Eq. (6).

6 $$Fit=min\left(\frac{\sum_{t=1}^{24}{P}_{LOSS-DG}(t)}{\sum_{t=1}^{24}{P}_{LOSS-base}(t)}\right) .$$

Here, ${P}_{LOSS-DG}$ and ${P}_{LOSS-base}$ are the active power loss with and without DG, respectively. The optimization of Eq. (6) is achieved by utilizing the proposed algorithm algorithm to meet the equality and inequality restrictions, as depicted in the following subsection.

### Network constraints

#### Power balance constraints

To keep the flow of active and reactive power in the system balanced, Eqs. (7) and (8)^34^ show the link between the power from the DG and the substation, as well as the system loss and the load demand.

7 $${P}_{sub}+\sum_{g=1}^{{n}_{dg}}{P}_{DG, g}= \sum_{h=1}^{n}{P}_{D,h}+\sum_{j=1}^{n-1}{P}_{l,j},$$

8 $${Q}_{sub}+\sum_{g=1}^{{n}_{dg}}{Q}_{DG,g}= \sum_{h=1}^{n}{Q}_{D,h}+\sum_{j=1}^{n-1}{Q}_{l,j},$$

where, ${P}_{sub}$ and ${Q}_{sub}$ are respectively corresponding to the active and reactive power injected by the grid. ${P}_{DG}$ and ${Q}_{DG}$, respectively, symbolize the active and reactive power supplied by the DGs. The total number of DGs and the reactive power loss are denoted by ${n}_{dg}$ and ${Q}_{l}$, respectively.

#### Inequality constraints

The capacity constraints of DG integrated into DN are given below:

9 $${P}_{DG, Total}\le \left({P}_{D, Total}+{P}_{PLOSS}\right) ,$$

10 $${Q}_{DG, Total}\le \left({Q}_{D, Total}+{Q}_{PLOSS}\right),$$

11 $${P}_{DG,min}\le {P}_{DG}\le {P}_{DG, max} ,$$

12 $${PF}_{DG, min}\le {PF}_{DG}\le {PF}_{DG, max},$$

where, ${PF}_{DG, min}$ and ${PF}_{DG, max}$ show the minimum and maximum boundaries of the OPF of the DG units, respectively. The upper and lower boundaries of the power generated by the DG is presented by ${P}_{DG,min}$ and ${P}_{DG,max}$, respectively. In this paper, the limitations of DG's active power exist within the range of $[0.3, 3]$ MW, while its power factor is within 0.7 and 1, respectively.

Connecting DGs to the grid will significantly impact the voltage at each bus. Hence, it is crucial to ensure that all node voltages remain within the defined limits^35,36^ and at the fundamental frequency.

13 $${V}_{j,min}\le {V}_{j}\le {V}_{j, max} .$$

Here, ${V}_{j,min}$ and ${V}_{j,max}$ are respectively the lower and upper voltage magnitudes at node $j$. These boundaries are chosen to be 5% of nominal values for satisfying good voltage stability.

**Figure 2:**
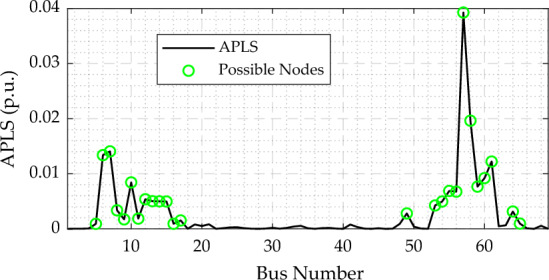
APLS index for the IEEE 69-bus distribution system.

Ultimately, the slack bus is excluded from the candidate nodes for integrating DGs; thus, the possible location for the DG allocation is constrained by Eq. (14), which follows^36^.

14 $${2\le Loc}_{DG}\le {n}_{c},$$

where, ${n}_{c}$ symbolizes the number of nodes that are candidates for DG installation. Note that, this paper utilizes the active power loss sensitivity (APLS) index to specify the feasible nodes for installing DGs to decrease the space of the search agents and decline the computational time of the optimizer, as depicted in Fig. 2, follows^10^. The possible nodes for installing DGs are approximately 36% of the system's buses.

### Calculation of performance indices

The impact of the optimal allocation of DGs on the performance of the DN is evaluated using four indices, as described below. These parameters comprise the total voltage deviation (TVD), summation voltage stability (SVS) index, power loss mitigation (PLM) index, and penetration level (PL) of the DGs.

The TVD is a crucial metric for determining the voltage quality; it is the aggregate of all the buses' voltage magnitudes relative to the swing bus voltage. Also, while assessing the reliability of a service, it is an essential metric to consider. Equation (15) can evaluate the TVD, follows^37^. In this equation, ${\text{V}}_{\text{j}}$, and ${\text{V}}_{\text{swing.}}$ refer to the $jth$ bus voltage magnitude and the swing bus voltage, respectively.

15 $$TVD=\sum_{j=1}^{n}\left|{V}_{swing.}-{V}_{j}\right|.$$

Moreover, it is critical to maintain a suitable range for the bus voltages at the distribution system level to prevent the occurrence of the earlier power system collapse. The voltage stability index (VSI) at bus $(s+1)$ can be measured using the following equation^38^:

16 $${VSI}_{\left(s+1\right)}={\left|{V}_{(s)}\right|}^{4}-4{\left({P}_{\left(s+1\right)}{X}_{\left(s,s+1\right)}-{Q}_{\left(s\right)}{R}_{\left(s,s+1\right)}\right)}^{2}-4{\left|{V}_{\left(s\right)}\right|}^{2}\left({Q}_{\left(s+1\right)}{X}_{\left(s,s+1\right)}+{P}_{\left(s+1\right)}{R}_{\left(s,s+1\right)}\right) .$$

Here, the real and reactive power at bus $s+1$ are denoted by ${P}_{\left(s+1\right)}$ and ${Q}_{\left(s+1\right)}$, respectively; ${V}_{(s)}$ refers to the voltage at bus $s$. Thus, the SVS index for the whale number of the system's bus is given below.

17 $$SVS=\sum_{k=1}^{n}{VSI}_{k} .$$

Equation (18) determines the PLM index, which is used to assess the percent reduction in power loss after deployment of DGs.

18 $$PLM=\frac{{P}_{LOSS-base}-{P}_{LOSS-DG}}{{P}_{LOSS-base}} \times 100.$$

Ultimately, the penetration level (PL) of the DGs, considering different technologies, is given by Eq. (19)^39^. In this formula, the total active power of the DG is represented by ${P}_{DG}$, while ${P}_{D}$ refers to the total demand of the system.

19 $$PL=\frac{{P}_{DG}}{{P}_{D}} \times 100 .$$

## Probabilistic models of generation and demand

In this section, we present the mathematical representation of the power produced by wind turbines and photovoltaic systems considering the uncertain nature of solar irradiation and wind speed. Additionally, we incorporate the time-varying characteristics of loads to account for the complete uncertainties associated with generation and demand.

### Photovoltaic modelling

The solar radiation variation throughout the day can be represented using the beta probability distribution function (BetaPDF) based on historical data gathered over a specific time period. To achieve this, the daily data is divided into 24-h segments, and the hourly mean and standard deviation (std) of radiation data are used. By discretizing the solar radiation values of each time segment into 20 states with a step size of 0.05 kW/m^2^, the BetaPDF is employed to capture the distributions in a discrete form. Subsequently, the expected power output per hour can be calculated. Recent studies, such as^40,41^, have utilized the beta distribution to model the intermittent characteristics of solar radiation, as demonstrated in Ref.^42^. The probability description of solar radiation can be expressed as follows.

20 $${P}_{\beta }\left(S\right)=\left\{\begin{array}{ll}\frac{\Gamma \left(\varphi +\varepsilon \right)}{\Gamma \left(\varphi \right)+\gamma \left(\varepsilon \right)}{s}^{\varphi -1}{(1-S)}^{\varepsilon -1} & \quad 0\le s\le 1, \varepsilon ,\varphi \ge 0 \\ 0 & \quad \text{ else}\end{array}\right.$$

Here, ${\text{P}}_{\upbeta }\left(\text{S}\right)$ symbolize the beta distribution function of $S$, while $\Gamma$ represents the gamma function. $\text{S}$ signifies a random variable of solar radiation in kW/m^2^. The mean $(a)$ and std $(b)$ of the solar radiation are mainly adopted to form the shape of the beta distribution, as per Eqs. (21) and (22)^42^.

21 $$\varphi =\left(1-a\right)\left(\frac{\Gamma \left(1+a\right)}{{b}^{2}}-1\right) ,$$

22 $$\varepsilon =\frac{a\varphi }{1-a},$$

The probability of solar radiation at a particular time may be specified using the aggregate distribution function (${\rho }_{s}$), as depicted in expression (23). While, ${S}_{1}$ and ${S}_{2}$ represent the constraints of the solar radiation state.

23 $${\rho }_{s}=\underset{{s}_{1}}{\overset{{s}_{2}}{\int }}{P}_{\beta }\left(S\right)dS.$$

The ambient temperature and solar radiation of the location are the principal governing parameters that influence the power produced by the PV module. The generated output power of the PV module, ${P}_{pv}\left(S\right)$ at a specific solar irradiance $S$, can be provided by Eq. (24), follows^42^.

24 $${P}_{pv}\left(S\right)=FF\times N\times {I}_{i}\times {V}_{v},$$

25 $$FF=\frac{{I}_{mp}\times {V}_{mp}}{{I}_{sc}\times {V}_{oc}},$$

26 $${V}_{v}={V}_{oc}-{T}_{c}\times {K}_{v},$$

27 $${I}_{i}=S \left[{I}_{sc}+{K}_{i}\times \left({T}_{c}-25\right)\right],$$

28 $${T}_{c}={T}_{a}+S\left(\frac{{T}_{o}-20}{0.8}\right),$$

where, $FF$, $N$ symbolize the fill factor and the total number of PV modules, respectively. ${I}_{mp}$ and ${V}_{mp}$ are the corresponding current and voltage at maximum power, respectively, while ${I}_{sc}$ and ${V}_{oc}$ denote respectively the short circuit current and open circuit voltage. ${T}_{o}$ and ${T}_{a}$ are the normal operating temperature and ambient temperature in °C; ${K}_{i}$ and ${K}_{v}$ illustrate respectively the temperature coefficients of current and voltage in A/℃ and V/°C.

For a given period $t$, the predicted average power output of a photovoltaic system ${P}_{pv-net}(t)$, may be determined by the following equation.

29 $${P}_{pv-net}\left(t\right)=\underset{0}{\overset{t}{\int }}{P}_{pv}\left(S\right) {\rho }_{s} dS.$$

**Figure 3:**
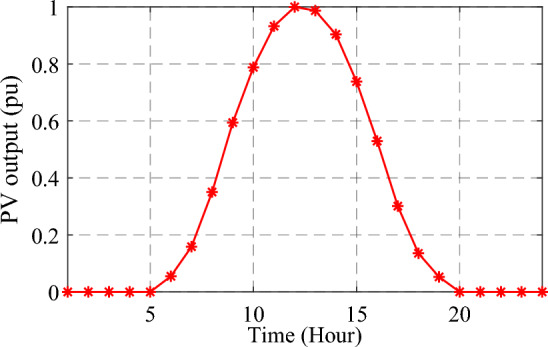
Normalized PV-generated power profile over 24 h.

**Figure 4:**
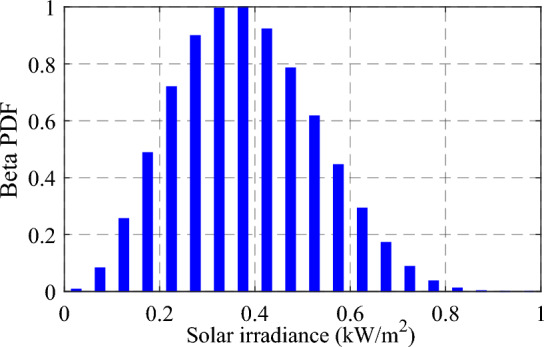
Beta distribution for the solar irradiance at 9.00 a.m.

Therefore, the normalized PV production for a day can be obtained by finding the average predicted power for each hour, as depicted in Fig. 3. Figure 4 clarifies the beta distribution representing the solar irradiance at 9.00 a.m., with a state value of 0.05 kW/m^2^.

### Wind turbine modelling

Weibull probability distribution function (WeibullPDF) has been implemented^43,44^ to characterize the probabilistic nature of wind speed over a certain time interval. For a given wind speed $v(m/s)$ at the $t$-time, the Weibull distribution ${W}_{v}\left(v\right)$, may be given as.

30 $${W}_{v}\left(v\right)=\frac{k}{c}{\left(\frac{v}{c}\right)}^{k-1}\text{exp}\left(-{\left(\frac{v}{c}\right)}^{k}\right) \forall c>1; v>0,$$

The following relations, Eqs. (31) and (32) describe the scale factor $(c)$ and shape parameter $(k)$ at a t-time interval, which are dependent on the mean ($\mu$) and std ($\sigma$) of wind speed.

31 $$k={\left(\frac{\sigma }{\mu }\right)}^{-1.086},$$

32 $$c=\frac{\mu }{\Gamma \left(1+\frac{1}{k}\right)}.$$

**Figure 5:**
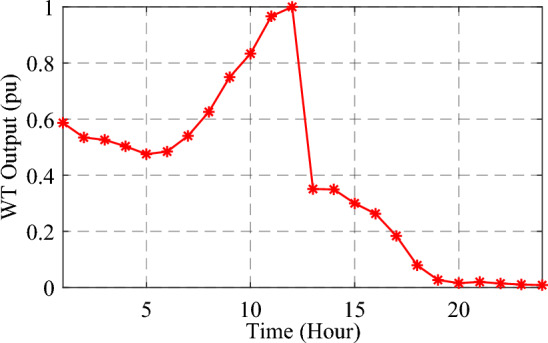
Normalized WT-generated power profile for 24 h.

As mentioned, to determine the amount of energy generated by DGs powered by the WT technology, the continuous PDF for a certain period has been partitioned into states (intervals) in which the wind velocity fall within predetermined restrictions^45^. In this study, each time segment of wind speed has been divided into 15 states with a step of 1 m/s, and the probability of all these possible states governs the power produced by the WT for that time. Hence, the average power of WT per hour $({P}_{wt})$ corresponding to a particular t-time interval, can be expressed by Eq. (33). Figure 5 shows a typical day for one year of the normalized output power of WT.

33 $${P}_{wt}=\sum_{i=1}^{{N}_{s}}{P}_{wt}\left({v}_{i}\right)*{F}_{w}\left({v}_{i}\right).$$

**Figure 6:**
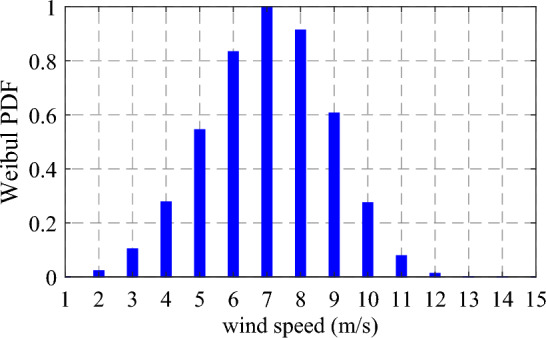
The WeibullPDF of wind speed at 7 a.m.

Here, ${N}_{s}$ represents the total number of wind speed discrete levels. The probability of the wind speed $({F}_{w}\left(v\right)$) for every level at any certain period $t$, is computed as Eq. (34). The WeibullPDF of wind speed at 7 a.m. is represented in Fig. 6.

34 $${F}_{w}\left(v\right)=\underset{{v}_{1}}{\overset{{v}_{2}}{\int }}{W}_{v}\left(v\right) dv ,$$

and the output power of WT with the average wind speed of ${v}_{avg}$, for a stage $i$, is formulated as per Eq. (35).

35 $${P}_{wt}\left(v\right)=\left\{\begin{array}{ll}0 & \quad {v}_{cout} <{v}_{avg}<{v}_{cin}\\ {K}_{1}\times {v}_{avg}^{3}+{K}_{1}\times {P}_{n} & \quad {v}_{cin}<{v}_{avg} < {v}_{n} \\ {P}_{n} & \quad {v}_{n}\le {v}_{avg}\le {v}_{cout}\end{array}\right.$$

where, ${P}_{n}$ and ${v}_{cout}$ identify, respectively, the nominal rated power of wind turbine and cut-out wind speed. ${K}_{1}$ and ${K}_{2}$ are constants that mainly depend on the cut-in speed ${(v}_{cin})$ and nominal wind speed $({v}_{n})$, respectively, and are formulated as^43^.

36 $${K}_{1}=\frac{{P}_{n}}{\left({v}_{n}^{3}-{v}_{cin}^{3}\right)},$$

37 $${K}_{2}=\frac{{v}_{cin}^{3}}{\left({v}_{n}^{3}-{v}_{cin}^{3}\right)}.$$

### Load modelling

**Figure 7:**
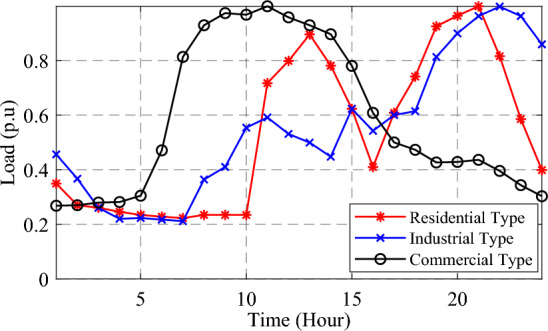
Commercial, residential, and industrial load patterns in p.u. for 24 h.

This paper addresses the time-variant load demands in the distribution networks, considering the need to supply dynamic loads. It examines industrial, residential, and commercial dynamic load models to identify the appropriate location and capacity of DGs using an analytical approach, as described in Ref.^9^. Figure 7 illustrates the normalized load pattern for one day, showcasing the maximum demand of 1 p.u., for these three types of demands. Equations (38) and (39) in Ref.^46^ express the mathematical representation of voltage-dependent dynamic load patterns during a specific time.

38 $${P}_{g}^{\prime}\left(t\right)={P}_{g}\left(t\right)\times {{V}_{g}}^{{e}_{ac}} ,$$

39 $${Q}_{g}^{\prime}\left(t\right)={Q}_{g}\left(t\right)\times {{V}_{g}}^{{e}_{rc}}.$$

Here, ${P}_{g}\left(t\right)$ and ${P}_{g}\left(t\right)$ are respectively the active and reactive power demand at bus $g$, while $Q^{\prime}_{g} \left( t \right)$ and $P^{\prime}_{g} \left( t \right)$ denote the injected reactive and real power at node $g$. The exponential reactive and real load voltages are symbolized by ${e}_{rc}$ and ${e}_{ac}$, respectively. The exponential coefficient $({e}_{ac})$ value is 0.920, 6.0, and 3.40 for residential, industrial, and commercial demand patterns, respectively. Similarly, The value of the exponential coefficient $({e}_{rc})$ is 4.040, 0.180, and 1.510 for residential, industrial, and commercial demand patterns, respectively.

## Optimization technique

### Original prairie dog optimizer

The prairie dog optimization is a metaheuristic algorithm inspired by the behavior of prairie dogs in nature. This section presents the mathematical formulas of PDO along with its optimization techniques. In nature, prairie dogs engage in various activities throughout the day, such as eating, evading predators, creating and maintaining holes, and searching for food^31^. They exhibit caution when foraging to avoid predators and use signals and sounds to communicate with other individuals. These signals convey information about the presence of predators, food availability, and more. Notably, prairie dogs can recognize different predators and their hunting patterns.

To mathematically model prairie dog behavior, certain assumptions are considered:

- The population consists of a certain number of prairie dogs ${n}_{p}$ organized into cliques, with a total of $m$ cliques.
- The entire population is divided into multiple precincts, with dogs from the same clique being assigned to the same precinct.
- Initially, ten holes are required in each precinct, and this number increases to 100 over time.
- Two types of signals are used: one indicating a new food source and the other signaling anti-hunting measures.
- The exploration phase involves searching for food and building holes, while the exploitation phase focuses on anti-hunting operations.
- Similar activities are performed by other cliques, and the solution space is divided into precincts (cliques). The exploitation and exploration operations are repeated for the number of cliques present.

The PDO optimizer starts with random population in the initial generation, similar to all other metaheuristics algorithms. Assume, the number of dogs is ${n}_{p}$ in the same coterie, then the location of ${i}^{th}$ in every coterie, which has a total number of $m$, can be given by the following equation.

40 $$CT= \left(\begin{array}{cc}\begin{array}{ccc}{CT}_{\text{1,1}}& {CT}_{\text{1,2}}& \dots \\ {CT}_{\text{2,1}}& {CT}_{\text{2,2}}& \dots \end{array}& \begin{array}{cc}{CT}_{1,d-1}& {CT}_{1,d}\\ {CT}_{2,d-1}& {CT}_{2,d}\end{array}\\ \begin{array}{ccc}.& .& \dots \\ {CT}_{m,1}& {CT}_{m,2}& \dots \end{array}& \begin{array}{cc}.& .\\ {CT}_{m,d-1}& {CT}_{m,d}\end{array}\end{array}\right),$$

where ${CT}_{i,j}$ refers to the dimension number $j$ of the *i*th individual. The next equation gives the location of the dogs in the same coterie.

41 $$PD= \left(\begin{array}{cc}\begin{array}{ccc}{PD}_{\text{1,1}}& {PD}_{\text{1,2}}& \dots \\ {PD}_{\text{2,1}}& {PD}_{\text{2,2}}& \dots \end{array}& \begin{array}{cc}{PD}_{1,d-1}& {PD}_{1,d}\\ {PD}_{2,d-1}& {PD}_{2,d}\end{array}\\ \begin{array}{ccc}.& .& \dots \\ {PD}_{{n}_{p},1}& {PD}_{{n}_{p},2}& \dots \end{array}& \begin{array}{cc}.& .\\ {PD}_{{n}_{p},d-1}& {PD}_{{n}_{p},d}\end{array}\end{array}\right).$$

The following two equations show the location for every coterie and every dog using uniform distribution.

42 $${CT}_{i,j}=U\left(\text{0,1}\right)\times \left({UB}_{j}-{LB}_{j}\right)+{LB}_{j },$$

43 $${PD}_{i,j}=U\left(\text{0,1}\right)\times \left({UB}_{j}-{LB}_{j}\right)+{LB}_{j,}$$

where ${UB}_{j},{LB}_{j}$ refer to upper and lower bounds respectively, $U$ is a uniformly distributed number with the interval [0, 1]. Equation (44) will be utilized to calculate the fitness function for all individuals and save them in an array.

44 $$f\left(PD\right)=\left[\begin{array}{l}{f}_{1}([{PD}_{\text{1,1}} {PD}_{\text{1,2}} \dots {PD}_{1,d-1} {PD}_{1,d}])\\ \begin{array}{l}{f}_{2}([{PD}_{\text{2,1}} {PD}_{\text{2,2}} \dots {PD}_{2,d-1} {PD}_{2,d}])\\ {f}_{.}([ . . \dots . . ])\end{array}\\ {f}_{{n}_{p}}([{PD}_{{n}_{p},1} {PD}_{{n}_{p},2} \dots {PD}_{{n}_{p},d-1} {PD}_{{n}_{p},d}])\end{array}\right].$$

#### Exploration phase

Exploration plays a vital role in every optimization algorithm, and the PDO incorporates exploration through the activities of food seeking and hole building. When prairie dogs face a scarcity of food in their current location, they instinctively move to new locations, thoroughly exploring the entire search space in search of nutrition resources or better solutions. The construction of holes is equally crucial for their survival, providing protection against predators and contributing to a healthy environment.

Prairie dogs typically reside in colonies that are divided into distinct areas or family units. Each group of prairie dogs explores and constructs holes within their designated boundaries. The exploration process occurs collectively, and they relocate to new locations only when there is a threat from predators. The PDO is divided into two phases: exploration and exploitation, which are based on four distinct scenarios. The total number of iterations is separated into four groups, with the first two categories using exploration and the following two categories employing exploitation. The first two parts utilized for exploration phase are applicable when $iter$ is less than $\frac{{Max}_{iter}}{4}$ or when $\frac{{Max}_{iter}}{4}$ is less than $\frac{{Max}_{iter}}{4}$ but less than $\frac{{Max}_{iter}}{2}$. The change in position for searching food and constructing new holes can be described by the following formulas:

45 $${PD}_{i+1,j+1}={GBest}_{i,j}-{eCBest}_{i,j}\times \rho -{CPD}_{i,j}\times Levy\left({n}_{p}\right) \quad \forall iter<\frac{{Max}_{iter}}{4}$$

46 $${PD}_{i+1,j+1}={GBest}_{i,j}\times rPD \times DS \times Levy\left({n}_{p}\right)\quad \forall \frac{{Max}_{iter}}{4}\le iter<\frac{{Max}_{iter}}{2}$$

where, ${GBest}_{i,j}$ signifies the best individual, ${eCBest}_{i,j}$ is the obtained best individual effects as shown in Eq. (47), ${CPD}_{i,j}$ refers to the effect of randomized cumulative as expressed in Eq. (48), $DS$ refers to the strength of coterie’s digging, and $rPD$ is the location of random individual, the specific food source alert $(\rho )$ has a set frequency of 0.1 kHz, $Levy$ is a leavy distribution, which is recognized to promote a more effective and efficient exploration of the issue search space.

47 $${eCBest}_{i+1,j+1}={GBest}_{i,j}\times \Delta +\frac{{PD}_{i,j} \times mean({PD}_{{n}_{p},m})}{{GBest}_{i,j}\times \left({UB}_{j}-{LB}_{j}\right)+\Delta } ,$$

48 $${CPD}_{i,j}=\frac{{GBest}_{i,j}-{rPD}_{i,j}}{{GBest}_{i,j}+\Delta },$$

49 $$DS=1.5\times r\times {\left(1-\frac{iter}{{Max}_{iter}}\right)}^{\left(2\frac{iter}{{Max}_{iter}}\right)} .$$

In the above equations, $r$ refers to random property for guaranteeing exploration and $\Delta$ refers to minor value which calculates difference that exist in the prairie dogs.

#### Exploitation phase

**Figure 8:**
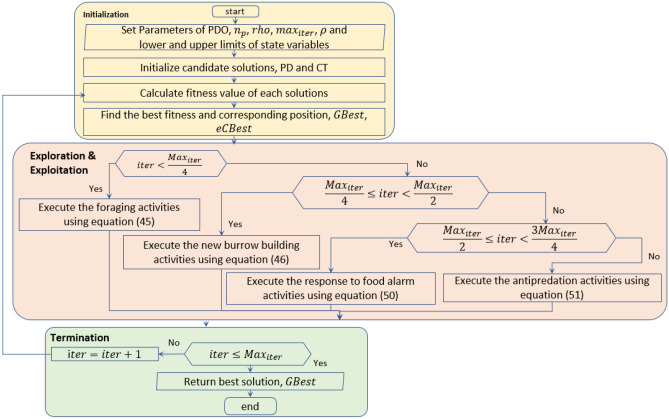
Flowchart of the original PDO algorithm.

This section focuses on the exploitation behavior in PDO. The exploitation phase of the optimization process utilizes the response of prairie dogs to two distinct signals. Prairie dogs use different sounds to communicate with each other in various situations, such as signaling the availability of food or the presence of predators. These communication and interaction skills are crucial for meeting the nutritional requirements of the dogs and improving their defensive capabilities against predators. These behaviors are mathematically simulated through the expressions given in Eqs. (50) and (51). Additionally, the flowchart of the original PDO is illustrated in Fig. 8.

50 $${PD}_{i+1,j+1}={GBest}_{i,j}-{eCBest}_{i,j}\times \tau -{CPD}_{i,j}\times rand \quad \forall \frac{{Max}_{iter}}{2}\le iter<3\frac{{Max}_{iter}}{4}$$

51 $${PD}_{i+1,j+1}={GBest}_{i,j}\times PE \times rand \quad \forall 3\frac{{Max}_{iter}}{4}\le iter<{Max}_{iter}$$

where, $PE$ refers to the impact of the predator and can be given in the following equation and $\tau$ signifies a small number indicating the quality of the food supply.

52 $$PE=1.5\times {\left(1-\frac{iter}{{Max}_{iter}}\right)}^{\left(2\frac{iter}{{Max}_{iter}}\right)}.$$

### Modified Prairie dog optimizer (mPDO)

The original PDO has limitations like imbalanced exploration and exploitation, susceptibility to local suboptimal regions, and premature convergence. To overcome these drawbacks, this study presents a new version of the prairie optimizer. Our proposed optimizer incorporates a novel exploration phase inspired by the slime mold algorithm (SMA)^47^ food approach. We enhance the explorative capability of mPDO by introducing the smell index parameter from SMA. The calculation of the smell index weight is as follows:

53 $$\overrightarrow{W(SmellIndex(i))}=\left\{\begin{array}{cc}1+r.\text{log}(\frac{GBest-SO(i)}{GBest-wf}+1)& condition\\ 1-r.\text{log}(\frac{GBest-SO(i)}{GBest-wf}+1)& others\end{array}\right.$$

where, $GBest$ refers to the best solution; $wf$ is the worst existed solution; $SO$ is the smell order of the sorted fitness function. We also introduce a non-linear operator called $A$, which calculated as follows:

54 $$A=\text{arctanh}\left(-\left(\frac{iter}{{max}_{iter}}\right)+1\right) .$$

Then, the novel exploration updating equation can be formulated as follows:

55 $${PD}_{i}=\left\{\begin{array}{ll}{GBest}_{i,j}+vb.(W*{PD}_{A}-{PD}_{B})& r<rand\\ {PD}_{A}+F.\propto . \left|{PD}_{A}-{PD}_{i}\right|& otherwisw\end{array}\right.$$

Here, $vb$ is a value which falls in the range $[-A,A]$ and $\propto$ can be calculated from $exp\left(\frac{-iter}{{max}_{iter}}\right),$ where $iter$, and ${max}_{iter}$ are respectively the current and total number of iterations, $F$ is a random variable falls in the rand [− 1, 1], and ${PD}_{A}, {PD}_{B}$ refer to two different randomly chosen individuals. Algorithm 1 provides in depth the pseudo code of the developed mPDO algorithm.

**Algorithm 1:**
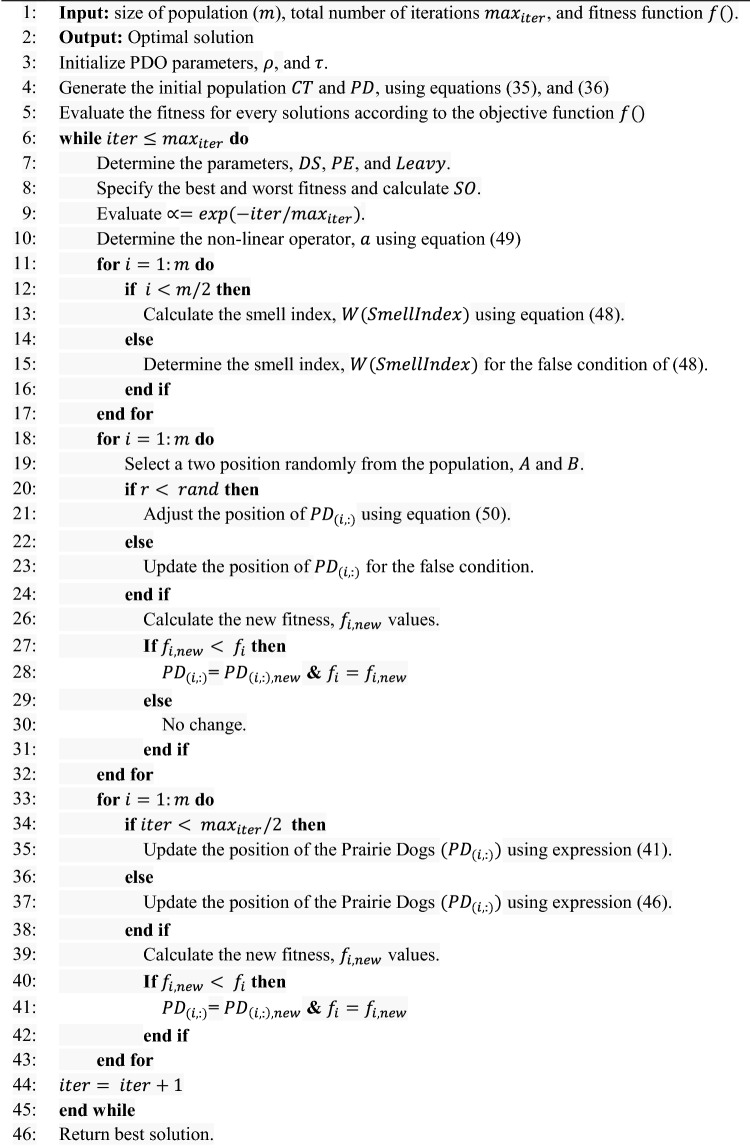
Pseudo-code of the mPDO algorithm.

## Numerical simulation and discussions

This section provides the mathematical validation of the modified algorithm compared with the other counterparts via cec2020 benchmark functions using different statistical metrics. Then, the mPDO is implemented to address the optimal inclusion of deterministic (the uncertainty associated with the generation and load demand is ignored) and stochastic (the uncertainty associated with the generation and load demand is considered) DGs in the 69-bus DN under numerous dynamic load behaviors. The main goal of the optimization model is to mitigate line loss in the system during the day.

### Experimental validation of mPDO algorithm

In this subsection, we assess the performance of the mPDO algorithm on 10 different functions from the cec2020 test suite benchmark, which involves unimodal, multimodal, hybrid, and composite functions. To ensure a fair comparison, we compare mPDO with the original PDO and eight other algorithms: COVID optimizer algorithm (COVIDIAO)^48^, Chernobyl disaster optimizer (CDO)^49^, tunicate swarm algorithm (TSA)^50^, sine cosine algorithm (SCA)^51^, stochastic paint optimizer (SPO)^52^, Harris hawks optimizer (HHO)^53^, particle swarm optimization (PSO)^54^, and whale optimization algorithm (WOA)^55^. The default parameters of these metaheuristic optimization algorithms are used.

Each algorithm is independently executed 30 times, with a maximum of 1000 iterations and a population size of 30, to ensure fair comparisons. Performance parameters in this study include minimum, maximum, mean, and std of fitness values, as well as Wilcoxon rank-sum p-values. The implementation of the developed optimizer is written in MATLAB R2020b M files and executed on a personal computer equipped with an Intel Core (TM) i7 G15-5511, processor running at 2.3 GHz and 16 GB of RAM.

#### Statistical results

This subsection presents the outcomes of the mPDO compared to other competitors in terms of different metrics, as previously mentioned. These comparisons are recorded in Table 1 for a dimension of 10. The table reveals that mPDO achieved the top rank in six functions (F2, F4, F6, F8-F10), ranked second in F5, and third in F3-F7. This demonstrates the superiority of the suggested optimizer.

**Table 1:** Results of the suggested optimizer with other 9 algorithms.

F	Criteria	mPDO	PDO	COVIDAO	CDO	TSA	WOA	SPO	HHO	PSO	SCA
F1	Min	1.910E + 02	1.463E + 10	1.668E + 10	2.093E + 10	2.980E + 03	1.463E + 07	2.910E + 03	2.910E + 03	2.900E + 03	3.062E + 03
	Max	1.237E + 04	3.191E + 10	2.824E + 10	2.172E + 10	5.037E + 03	7.191E + 07	3.200E + 03	3.011E + 03	3.000E + 03	3.641E + 03
	Avg	6.668E + 03	2.443E + 10	2.342E + 10	2.145E + 10	3.531E + 03	3.052E + 07	3.000E + 03	2.980E + 03	2.963E + 03	3.200E + 03
	STD	4.000E + 03	4.525E + 09	2.836E + 09	1.531E + 08	4.746E + 02	1.641E + 07	6.472E + 01	2.510E + 01	3.172E + 01	1.326E + 02
	Rank	6	10	9	8	5	7	3	2	**1**	4
F2	Min	1.736E + 03	4.114E + 03	3.910E + 03	4.454E + 03	2.763E + 03	2.494E + 03	1.487E + 03	2.046E + 03	1.900E + 03	4.292E + 03
	Max	3.383E + 03	6.164E + 03	5.657E + 03	5.342E + 03	5.700E + 03	5.089E + 03	6.037E + 03	3.836E + 03	3.756E + 03	5.593E + 03
	Avg	2.573E + 03	5.271E + 03	5.237E + 03	4.994E + 03	4.037E + 03	3.746E + 03	3.657E + 03	2.900E + 03	2.683E + 03	5.114E + 03
	STD	4.510E + 02	5.000E + 02	2.980E + 02	2.089E + 02	6.413E + 02	5.936E + 02	1.200E + 03	4.641E + 02	4.657E + 02	2.824E + 02
	Rank	**1**	10	9	7	6	5	4	3	2	8
F3	Min	7.400E + 02	9.114E + 02	1.000E + 03	9.694E + 02	8.746E + 02	8.463E + 02	7.421E + 02	8.356E + 02	7.413E + 02	8.746E + 02
	Max	9.283E + 02	1.172E + 03	1.104E + 03	1.023E + 03	1.046E + 03	1.046E + 03	9.062E + 02	9.712E + 02	7.792E + 02	1.011E + 03
	Avg	7.900E + 02	9.859E + 02	1.062E + 03	9.900E + 02	9.494E + 02	9.573E + 02	7.836E + 02	9.000E + 02	7.593E + 02	9.315E + 02
	STD	4.792E + 01	5.900E + 01	2.283E + 01	1.308E + 01	4.104E + 01	4.487E + 01	3.568E + 01	3.011E + 01	1.123E + 01	2.874E + 01
	Rank	3	8	10	9	6	7	2	4	**1**	5
F4	Min	1.900E + 03	5.944E + 04	3.700E + 04	1.463E + 05	2.123E + 03	1.923E + 03	1.900E + 03	1.910E + 03	1.900E + 03	2.000E + 03
	Max	1.900E + 03	1.500E + 06	5.213E + 05	3.910E + 05	6.593E + 05	2.089E + 03	2.712E + 04	1.936E + 03	1.910E + 03	6.037E + 03
	Avg	1.900E + 03	3.836E + 05	1.454E + 05	3.756E + 05	1.011E + 05	1.953E + 03	3.308E + 03	1.923E + 03	1.900E + 03	2.862E + 03
	STD	6.623E-01	3.683E + 05	9.554E + 04	5.531E + 04	1.675E + 05	3.700E + 01	4.944E + 03	5.263E + 00	8.862E-01	7.994E + 02
	Rank	**1**	10	8	9	7	4	6	3	2	5
F5	Min	2.443E + 04	7.157E + 05	7.773E + 05	4.510E + 05	9.971E + 04	7.200E + 04	6.604E + 03	4.573E + 04	1.953E + 04	1.413E + 05
	Max	4.593E + 05	1.454E + 07	5.052E + 06	2.315E + 06	4.893E + 06	5.037E + 06	2.191E + 07	2.213E + 06	2.593E + 05	3.593E + 06
	Avg	1.963E + 05	3.593E + 06	2.746E + 06	8.756E + 05	1.611E + 06	1.200E + 06	1.700E + 06	5.813E + 05	9.963E + 04	1.581E + 06
	STD	1.308E + 05	2.700E + 06	9.900E + 05	3.712E + 05	1.368E + 06	1.023E + 06	5.200E + 06	4.487E + 05	6.062E + 04	8.500E + 05
	Rank	2	10	9	4	7	5	8	3	**1**	6
F6	Min	1.635E + 03	2.487E + 03	2.694E + 03	2.763E + 03	2.157E + 03	1.792E + 03	1.623E + 03	1.784E + 03	1.724E + 03	2.145E + 03
	Max	2.164E + 03	3.841E + 03	3.454E + 03	3.472E + 03	2.792E + 03	2.994E + 03	2.712E + 03	2.724E + 03	2.263E + 03	2.784E + 03
	Avg	1.792E + 03	3.046E + 03	3.074E + 03	3.093E + 03	2.454E + 03	2.421E + 03	1.994E + 03	2.164E + 03	1.936E + 03	2.443E + 03
	STD	1.342E + 02	3.525E + 02	2.011E + 02	1.712E + 02	1.936E + 02	2.910E + 02	2.568E + 02	2.123E + 02	1.315E + 02	1.494E + 02
	Rank	**1**	8	9	10	7	5	3	4	2	6
F7	Min	8.531E + 03	2.443E + 05	1.421E + 05	4.164E + 05	1.413E + 04	1.011E + 04	4.052E + 03	6.157E + 04	4.683E + 03	6.172E + 04
	Max	5.900E + 05	1.104E + 07	3.213E + 06	1.500E + 08	1.123E + 07	3.581E + 06	6.052E + 05	8.263E + 05	3.134E + 05	1.454E + 06
	Avg	2.271E + 05	2.859E + 06	7.494E + 05	2.635E + 07	1.292E + 06	1.011E + 06	1.191E + 05	2.421E + 05	8.923E + 04	5.256E + 05
	STD	1.675E + 05	2.694E + 06	6.023E + 05	3.256E + 07	2.368E + 06	9.807E + 05	1.510E + 05	1.756E + 05	7.953E + 04	4.023E + 05
	Rank	3	9	6	10	8	7	2	4	**1**	5
F8	Min	2.308E + 03	3.145E + 03	4.335E + 03	4.213E + 03	2.433E + 03	2.326E + 03	2.308E + 03	2.315E + 03	2.308E + 03	2.611E + 03
	Max	5.568E + 03	7.074E + 03	5.792E + 03	7.335E + 03	6.554E + 03	6.736E + 03	7.472E + 03	6.089E + 03	5.980E + 03	7.172E + 03
	Avg	3.114E + 03	5.227E + 03	5.263E + 03	5.494E + 03	4.746E + 03	3.963E + 03	3.487E + 03	4.062E + 03	3.200E + 03	4.862E + 03
	STD	1.292E + 03	1.023E + 03	3.000E + 02	1.421E + 03	1.421E + 03	1.700E + 03	1.712E + 03	1.593E + 03	1.335E + 03	1.910E + 03
	Rank	**1**	8	9	10	6	4	3	5	2	7
F9	Min	2.813E + 03	3.052E + 03	3.172E + 03	3.326E + 03	2.953E + 03	2.900E + 03	2.554E + 03	2.923E + 03	2.836E + 03	2.971E + 03
	Max	2.874E + 03	3.191E + 03	3.342E + 03	3.400E + 03	3.247E + 03	3.093E + 03	3.335E + 03	3.335E + 03	2.923E + 03	3.046E + 03
	Avg	2.841E + 03	3.145E + 03	3.283E + 03	3.368E + 03	3.123E + 03	3.000E + 03	3.052E + 03	3.114E + 03	2.862E + 03	3.000E + 03
	STD	1.487E + 01	3.443E + 01	4.074E + 01	2.046E + 01	6.510E + 01	5.472E + 01	1.356E + 02	9.200E + 01	2.525E + 01	2.052E + 01
	Rank	**1**	8	9	10	7	4	5	6	2	3
F10	Min	2.900E + 03	3.623E + 03	3.568E + 03	5.164E + 03	2.980E + 03	2.944E + 03	2.910E + 03	2.910E + 03	2.900E + 03	3.062E + 03
	Max	2.994E + 03	6.413E + 03	5.134E + 03	5.971E + 03	5.037E + 03	3.145E + 03	3.200E + 03	3.011E + 03	3.000E + 03	3.641E + 03
	Avg	2.936E + 03	4.792E + 03	4.413E + 03	5.910E + 03	3.531E + 03	3.046E + 03	3.000E + 03	2.980E + 03	2.963E + 03	3.200E + 03
	STD	2.736E + 01	7.454E + 02	3.736E + 02	1.433E + 02	4.746E + 02	4.443E + 01	6.472E + 01	2.510E + 01	3.172E + 01	1.326E + 02
	Rank	**1**	9	8	10	7	5	4	3	2	6

Furthermore, mPDO delivers competitive results when compared to other approaches and achieves optimal values for nearly all of the cec2020 test functions. Table 1 confirms that the mPDO approach surpassed the other efficient algorithms in terms of Friedman rank test results.

The mPDO shows notable strengths in several benchmark functions. For instance, it achieves the best performance (rank 1) in functions F2, F4, F6, F8, and F9 in terms of minimum values. This indicates that mPDO is particularly effective at finding optimal or near-optimal solutions in these cases, demonstrating its robustness and efficiency in various scenarios.

In terms of average performance, mPDO often ranks among the top performers. For example, in functions F2, F3, F6, F9, and F10, it consistently shows competitive average values, highlighting its ability to maintain a good balance between exploration and exploitation throughout the optimization process.

#### Convergence curve

**Figure 9:**
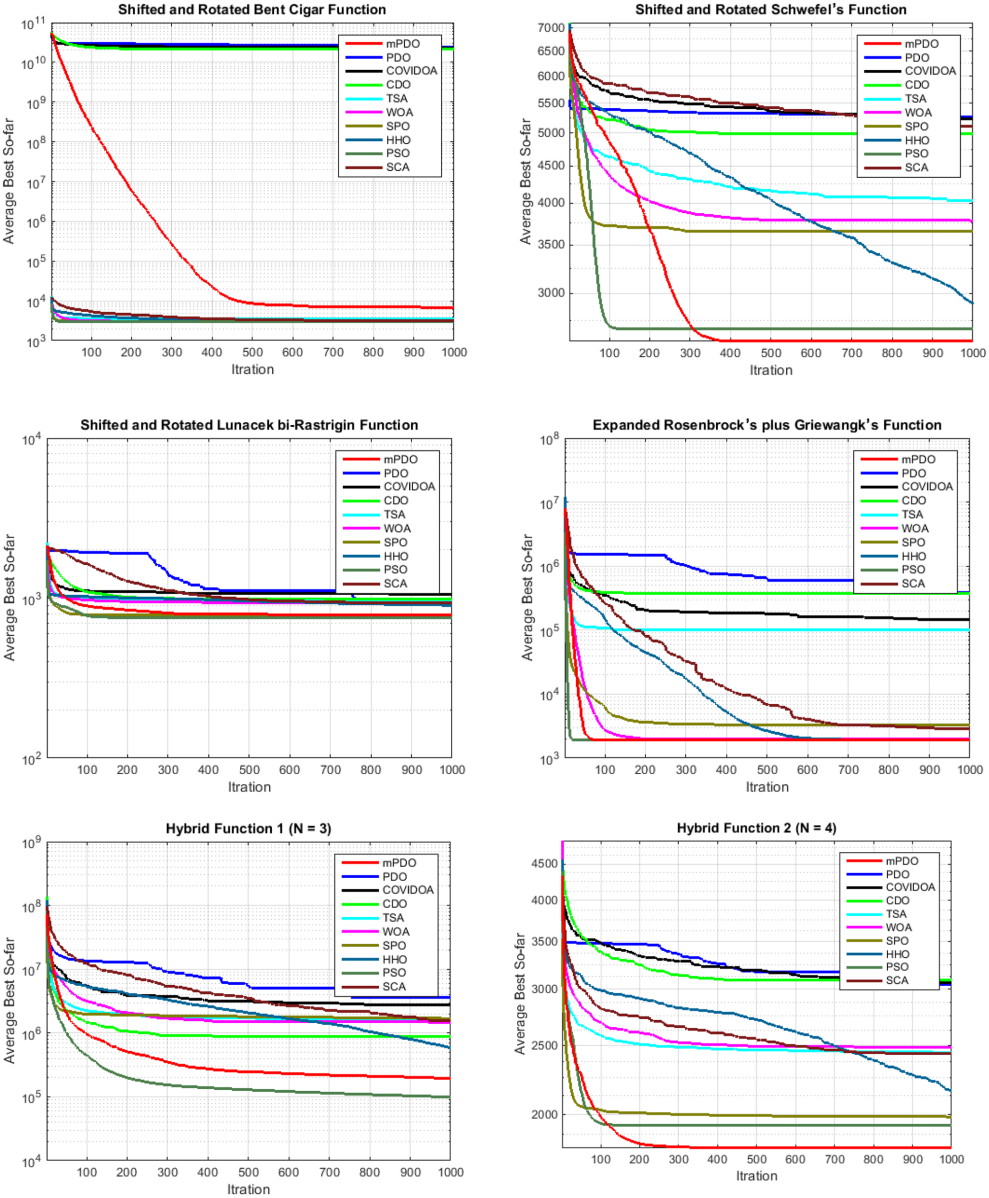

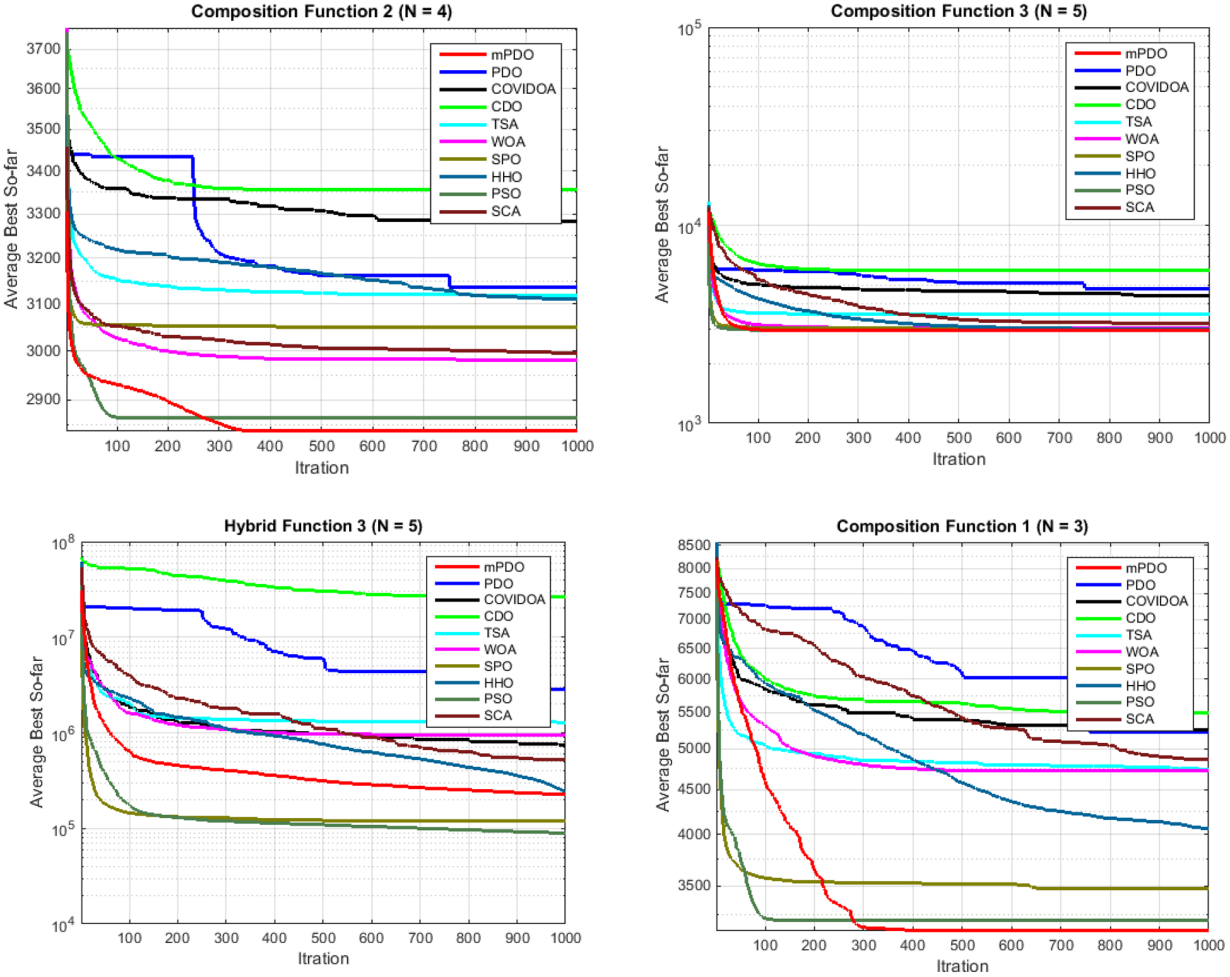
Convergence curve of mPDO with other competitors.

This subsection presents a graphical analysis of the convergence rate of mPDO across 10 functions from the cec2020 benchmark, with a dimension of 10. The convergence characteristics of all optimizers for these ten functions are depicted in Fig. 9. From this figure, it is evident that the proposed mPDO exhibits quick convergence rate compared to other methods, particularly for functions F2, F3, F4, F6, F8, F9, and F10. However, in certain cases such as functions F1, F5, and F7, mPDO becomes trapped in a near-optimal state. Nevertheless, it is worth noting that the suggested optimizer consistently achieves superior solutions in most cases, requiring fewer iterations. This rapid convergence property positions the mPDO algorithm as a viable tool for efficiently addressing online optimization issues that demand efficient calculation.

#### Boxplot behavior analysis

**Figure 10:**
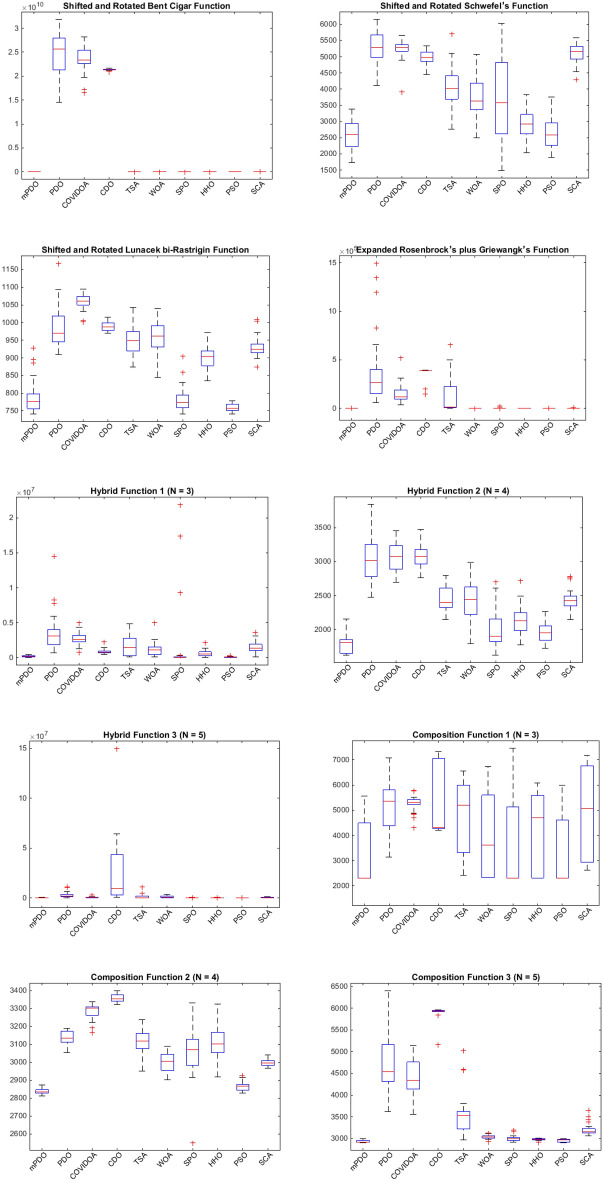
The curves of Boxplot curves for mPDO and other competitors optimizers over CEC0 2020 benchmark.

Figure 10 illustrates the boxplot curves representing the mPDO, as well as its competitors, showcasing data distributions across various functions from cec2020 with a dimension of 10. The boxplots depict the minimum and maximum values achieved by the algorithms, represented by the edges of the whiskers. A narrow boxplot indicates a high level of agreement within the data.

Analyzing the boxplots for the majority of functions, it is evident that the suggested mPDO approach exhibits the best distribution with lower values. Consequently, the mPDO algorithm outperforms other recent optimization algorithms in most cases, emphasizing its superior performance.

#### Wilcoxon rank sum test analysis

To validate the significance of the findings acquired by mPDO and other algorithms, a statistical test known as the Wilcoxon rank-sum test^56^ was conducted. This test was performed to demonstrate that the observed performance was not merely a result of chance. The results of the test, achieved at a significance level of 5% for most functions, are listed in Table 2. These results provide evidence that mPDO is a suitable and well-structured set of optimization rules, enabling the attainment of optimal solutions.

**Table 2:** Wilcoxon Rank sum test for mPDO vs compared algorithm cec2020.

mPDO vs	PDO	COVIDAO	CDO	TSA	WOA	SPO	HHO	PSO	SCA
F1	3.025E–11	3.025E–11	3.025E–11	5.324E–03	3.025E–11	2.257E–04	2.654E–04	1.111E–04	8.569E–04
F2	3.025E–11	3.025E–11	3.025E–11	6.725E–10	1.415E–09	1892E–04	1.232E–02	5.599E–01	3.025E–11
F3	4.081E–11	3.025E–11	3.025E–11	1.214E–10	1.333E–10	9477E–01	4.186E–09	4.432E–03	1.962E–10
F4	3.025E–11	3.025E–11	3.025E–11	3.025E–11	3.025E–11	4.501E–11	3.025E–11	8.652E–01	3.025E–11
F5	3.025E–11	3.025E–11	4.501E–11	2.000E–06	3.012E–07	2.163E–03	1.257E–05	3.501E–03	1.962E–10
F6	3.025E–11	3.025E–11	3.025E–11	3.341E–11	4.203E–10	1.302E–03	1.108E–08	7.203E–05	3.341E–11
F7	3.163E–10	8.847E–07	4.501E–11	3.401E–01	3.376E–04	1.068E–03	6.415E–01	4.232E–04	2.622E–03
F8	6.654E–07	3.268E–07	3.572E–06	2.687E–06	2.962E–05	1.068E–03	1.172E–05	3.631E–01	3.094E–06
F9	3.025E–11	3.025E–11	3.025E–11	3.025E–11	3.025E–11	5.572E–10	3.025E–11	1.432E–05	3.025E–11
F10	3.025E–11	3.025E–11	3.025E–11	3.341E–11	1.094E–10	1.163E–07	1.075E–07	1.864E–03	3.025E–11

### DG optimal allocation problem

**Figure 11:**
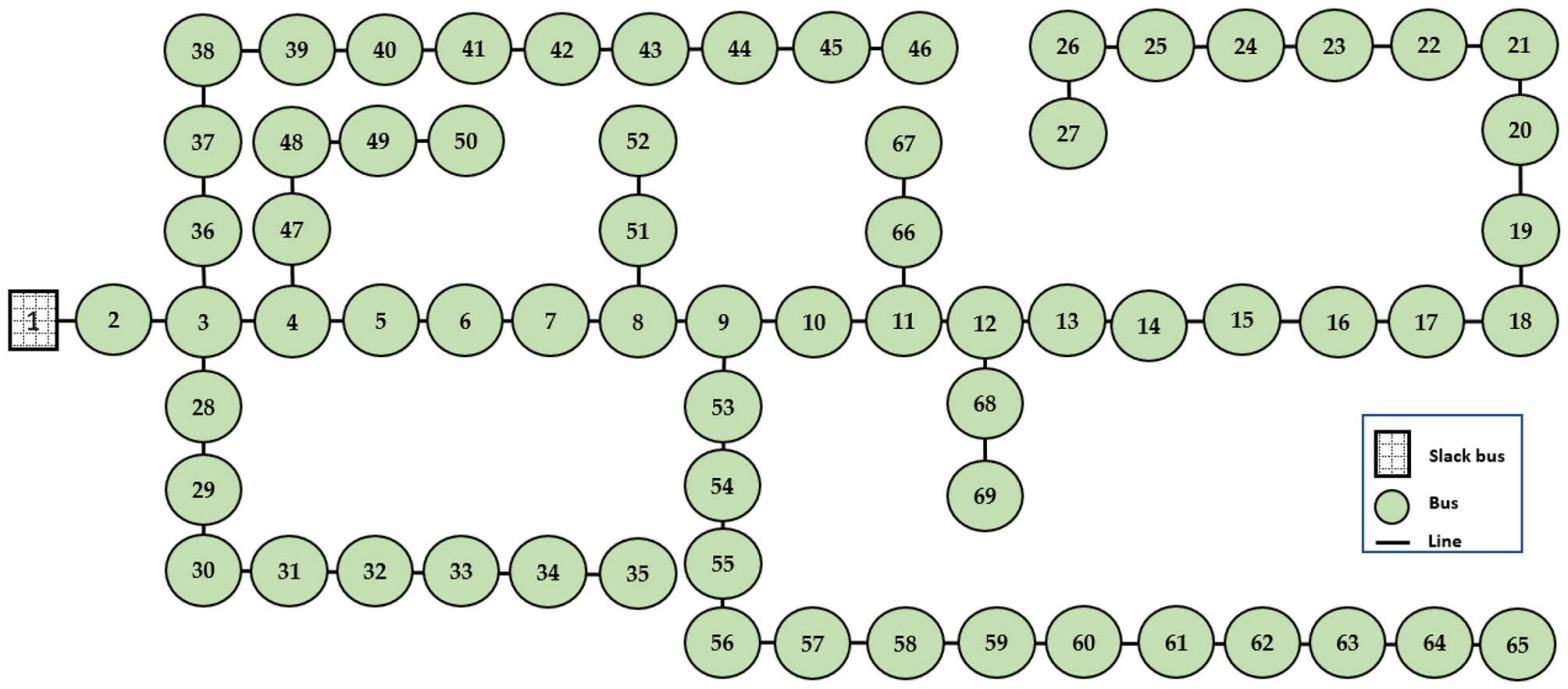
The configuration of the 69-bus radial distribution feeders.

In this subsection, the proposed mPDO algorithm is applied to investigate the impacts of different dynamic load patterns on the performance of the 69-bus DN (shown in Fig. 11) and the scheduling of the PV and WT units. This is achieved under deterministic and stochastic DG optimum allocation challenges. Moreover, a comparative study is performed to evaluate the performance of the mPDO against the traditional PDO and other robust optimization approaches under constant power load requirements. The overall load demand for this distribution system, which includes 69 nodes and 68 lines, is 3801.5 kW and 2694.6 kVAR^57^. The base MVA and kV are, respectively, 100 and 12.66. The summation of reactive and active line losses of the network before allocating renewable DGs are 102.16 kVAR and 225.0012 kW, respectively, while the lowest voltage profile, TVD, and SVS index are, respectively, 0.90919 p.u., 1.8374, and 61.2173.

The hourly historical statistics of solar radiation^58^ and wind speed^43^ are utilized to accommodate the periodic characteristics of renewable energy resources. In this study, the average values for the four seasons through 24 h are adopted to express the uncertainty throughout a one practical day. The total number of iterations and population size are 200 and 50 across all studied scenarios. In summary, this research studies many optimization situations as below:

Case #1: Deterministic optimal plaining of PV and WT.

Case #2: Stochastic optimal planning of PV and WT under commercial demand.

Case #3: Stochastic optimal planning of PV and WT under industrial demand.

Case #4: Stochastic optimal planning of PV and WT under residential demand.

Case #5: Stochastic optimal planning of PV and WT under mixed demand.

Case #6: Synchronous optimum PV and WT allocation for the different load models.

#### Case#1: deterministic optimal plaining of PV and WT

Table 3 presents the findings of the mPDO for providing the optimal capacity and placement of single and multiple PV in the 69-bus system. The results are compared with the traditional PDO and other algorithms reported in the literature, assuming no hourly variation in solar radiation. In this instance, the PVs contribute only active power to the grid i.e., operate at UPF.

**Table 3:** Numerical simulations of the mPDO and other algorithms for incorporating PV into 69-bus DN without uncertainty.

DGs units	Technique	Active loss	PLMI	Location	Size (kW)		SVS	TVD
		(kW)	(%)		Unit	Net capacity	(p.u.)	(p.u.)
	Without DG	225.0012	–	–	–	–	61.2173	1.8374
One PV	mPDO	83.2231	63.0122	61	1872.7055	1872.7055	64.6165	0.8729
	PDO	83.2269	63.0105	61	1862.2538	1862.2538	64.5979	0.8779
	BA^59^	83.2200	63.0135	61	1900	1900	–	–
	NHA^5^	83.3000	62.9780	61	1823	1823	–	–
	PSO^59^	83.2200	63.0135	61	1900	1900	–	–
Two PVs	mPDO	71.6750	68.1446	17	531.4831	2312.9532	66.0244	0.5002
				61	1781.4701			
	PDO	72.0215	67.9906	17	457.6431	2190.1964	65.717	0.5801
				61	1732.5533			
	BA^59^	73.30	67.42	17	500	2500	–	–
				61	2000			
	NHA^5^	71.80	68.0891	17	520	2253	–	–
				61	1733			
	PSO^59^	73.10	67.5113	15	400	2300	–	–
				61	1900			
Three PVs	mPDO	69.4272	69.1436	11	526.6687	2626.1494	66.2205	0.4498
				17	380.5104			
				61	1718.9703			
	PDO	70.4844	68.6738	12	309.4201	2401.1164	65.8738	0.5393
				16	306.1469			
				61	1785.5494			
	BA^59^	72.60	67.7335	13	400	2700	–	–
				22	300			
				61	2000			
	SFSA^6^	69.428	69.1433	11	527.3	2627.6	–	–
				18	380.5			
				61	1719.8			
	NHA^5^	69.70	69.0224	12	471	2472	–	–
				21	312			
				61	1689			
	QOCSOS^29^	69.4284	69.14	11	526.9	2626.2	–	–
				18	380.3			
				61	1719.0			
	CQOBMO_7^30^	70.05	68.86	11	573.02	2511.72	–	–
				18	355.20			
				61	1583.50			
	IGJO^60^	69.4272	69.1436	11	526.6687	2626.1495	–	–
				17	380.5104			
				61	1718.9703			

The table demonstrates that the active power loss is reduced by 63.0122%, 68.1446%, and 69.1436% when employing one, two, and three PV units, respectively, in the test network. Furthermore, the corresponding TVD decreases from the base value of 1.8374 p.u. to 0.8729 p.u., 0.5002 p.u., and 0.4498 p.u. with the incorporation of one, two, and three PV units, respectively. Additionally, installing one, two, and three PV units enhances the SVS from the base value of 61.2173 p.u. to 64.6165 p.u., 66.0244 p.u., and 66.2205 p.u., respectively. It is worth noting that the number of state variables in this case increases to six when three units of PV systems are installed, considering the location and capacity variables.

**Figure 12:**
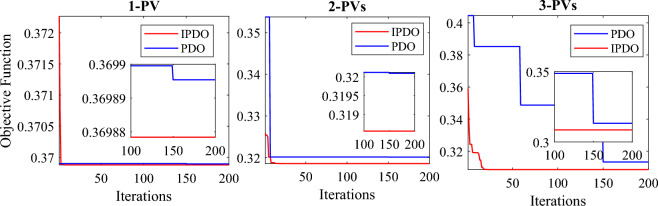
Convergence curves of the mPDO compared with the PDO for optimal allocating PVs in the 69-bus system without uncertainty.

To assess the efficiency of the mPDO, it is compared with the original PDO, bat algorithm (BA)^59^, PSO^59^, improved golden jackal optimization (IGJO)^60^, novel heuristic approach (NHA), novel stochastic fractal search algorithm (SFSA), quasi-oppositional-chaotic symbiotic organisms search algorithm (QOCSOS)^29^, and chaotic quasi-oppositional barnacles mating optimizer (CQOBMO-7)^30^. It is observed from Table 3 that the mPDO consistently beats the basic PDO and the state-of-the-art optimizers, exhibiting the lowest power loss when incorporating single and multiple PVs into the test system. This also demonstrated by Fig. 12, which illustrates convergence performance of the mPDO and the original algorithm. Accordingly, maximizes the technical benefits of the PV-based DG system.

As indicated in Tables 3 and 4, the WT has a more favorable impact compared to PV systems because it can contribute both active and reactive power to the grid. In this scenario, the mPDO achieves significant reductions in active power loss when integrating one, two, or three WT units into the test network, with percentages of 89.7028%, 96.7994%, and 98.1039% respectively. Incorporating one, two, or three WT units with OPF also results in a decrease in TVD from the base value of 1.8374 per unit (p.u.) to 0.5873 p.u., 0.1300 p.u., and 0.06449 p.u. respectively. Furthermore, the SVS index increases from 61.2173 p.u. (the base value) to 65.7205 p.u. (for one WT), 67.4823 p.u. (for two WTs), and 67.7427 p.u. (for three WTs). It is worth noting that the number of state variables raises to nine when three WTs are installed, considering the variables of location, size, and power factor.

**Table 4:** Numerical simulations of the mPDO and other algorithms for incorporating WT into 69-bus DN without uncertainty.

DGs units	Technique	Active loss	PLMI	Location	Size (kW)		SVS	TVD
		(kW)	(%)		Unit	OPF	(p.u.)	(p.u.)
	Without DG	225.0012	–	–	–	–	61.2173	1.8374
One WT	mPDO	23.1688	89.7028	61	1828.4670	0.8149	65.7205	0.5873
	PDO	23.1698	89.7024	61	1833.9214	0.8157	65.7305	0.5847
	BA^59^	52.50	76.6668	61	2100	0.98	–	–
	HSSA^61^	23.1688	89.7028	61	2243.9	0.82	–	–
	PSO^59^	52.50	76.6668	61	2100	0.98	–	–
Two WTs	mPDO	7.2013	96.7994	17	522.3446	0.8282	67.4823	0.1300
				61	1734.6740	0.8139		
	PDO	7.6200	96.6134	17	445.4596	0.7840	67.4146	0.16399
				61	1823.1392	0.8270		
	BA^59^	38.70	82.8001	17	600	0.98	–	–
				61	2000	0.98		
	HSSA^61^	7.2013	96.7994	17	630.7	0.83	–	–
				61	2131.3	0.81		
	PSO^59^	41.10	81.7334	18	600	0.98	–	–
				61	2200	0.98		
Three WTs	mPDO	4.2664	98.1039	11	494.3907	0.8133	67.7427	0.06449
				17	379.2248	0.8333		
				61	1674.3292	0.8138		
	PDO	5.5716	97.5237	7	416.4421	0.7035	67.6581	0.09135
				17	457.1182	0.8283		
				61	1716.7331	0.8228		
	SFSA^6^	4.2980	98.09	11	566.9	0.8190	–	–
				21	336.0	0.8330		
				61	1675.2	0.8180		
	QOCSOS^29^	4.2674	98.10	11	608.1	0.813	–	–
				18	454.9	0.833		
				61	2057.3	0.814		
	HSSA^61^	4.269	98.1027	11	608.9	0.82	–	–
				17	454.9	0.84		
				61	2057.1	0.81		
	PSO^59^	39.20	82.5779	61	1500	0.98	–	–
				59	600	0.98		
				16	500	0.98		
	IGJO^60^	4.2664	98.1038	11	492.2100	0.8108	–	–
				17	380.3056	0.8347		
				61	1675.1380	0.8141		

**Figure 13:**
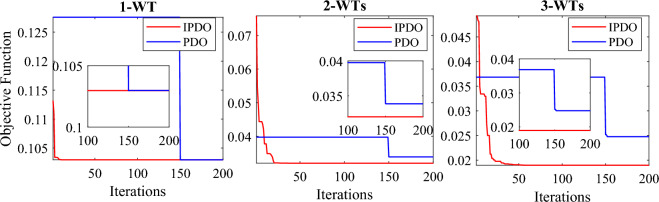
Convergence curves of the mPDO compared with the PDO for optimal allocating WTs in the 69-bus system without uncertainty.

The efficacy of the mPDO algorithm is evaluated by comparing its results with those obtained from the traditional PDO, BA, PSO, NHA, SFSA, QOCSOS, and hybrid symbiotic organisms search algorithm (HSSA)^61^. Table 2 demonstrates that the integration of single and multiple PV units into the DN leads to considerable decline in power loss when using the mPDO, surpassing the performance of other rivals. Figure 13 illustrates the convergence behavior of both the improved and traditional PDO methods.

In summary, the mPDO exhibits faster and more stable convergence towards the optimal solution, as depicted in Figs. 12 and 13. Moreover, considering the ability of WT to inject reactive power, the integration of WT yields superior results compared to PV in terms of power loss reduction, voltage profile enhancement, and voltage stability improvement. Therefore, only mPDO is utilized in the subsequent sections to determine the optimal positioning and sizing of single and multiple PV and WT units integrated into the distribution system. This is done to reduce power loss per day while considering the stochastic nature of solar irradiance, variation in wind speed, and time-varying load demand patterns, including commercial, residential, and industrial demands.

#### Case #2: stochastic optimal planning of PV and WT under commercial demand

**Figure14:**
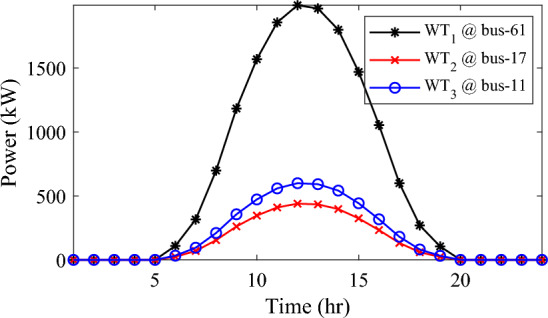
PV output power at nodes11, 17 and 61 using the mPDO algorithm during installing three PVs for the commercial load.

**Figure 15:**
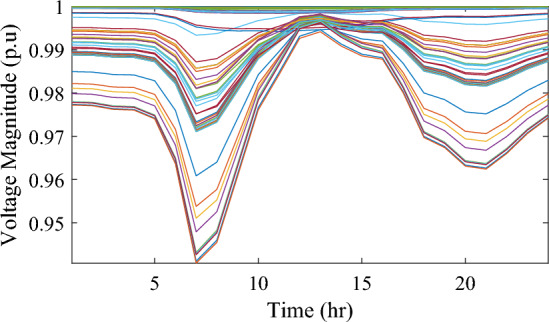
Voltage profile of 69-bus DN during integrating three PVs for the commercial load type.

In this particular scenario, the total reactive load profile throughout 24 h amounts to 37,818.4235 kVAr, while the total active load demand is 53,353.5065 kW. Without the integration of WTs or PVs into the test system, the total reactive and active loss over 24 h is 491.2141 kVAr, and 2,173.9944 kW, respectively. However, by incorporating a single PV unit at node 61 using the mPDO, the active power loss of the system is significantly reduced to 1,124.5492 kWh for 24 h, achieving a 48.2727% reduction. Moreover, the optimal placement and sizing of two PV units at nodes 61 and 17 result in a system loss of 1,038.2363 kW, representing a further 4% improvement compared to the integration of a single PV unit. Furthermore, the deployment of three PVs at nodes 11, 17, and 61, with a net sizing of 3,030.4973 kW, alleviated the power loss to 1,021.7016 kW, achieving a slight additional reduction of 53.0035% compared to the two-PV configuration. The total energy generated by incorporating one, two, and three PV units into the test network amounts to 16,319.1394 kWh, 20,130.4336 kWh, and 22,808.8910 kWh, respectively. Figure 14 illustrates the total daily energy output when three PV units are integrated at nodes 11, 17, and 61, operating at UPF. The voltage profiles of the present system are graphically depicted in Fig. 15, revealing that the optimal sitting of three PVs is unable to meet the minimum voltage constraints. For instance, bus 65 exhibits the smallest voltage levels of approximately 0.9407 p.u at 7 a.m. and 0.9455 p.u at 8 a.m.

**Figure 16:**
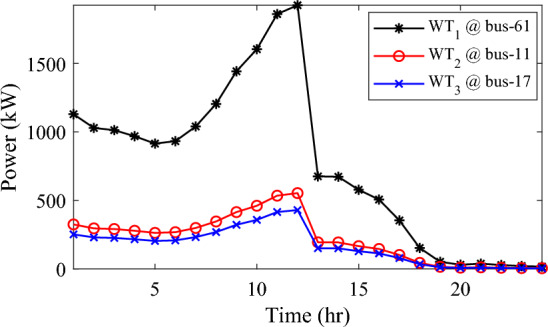
WT output power at nodes11, 17 and 61 using the mPDO algorithm during installing three WTs for the commercial load.

Alternatively, integrating one, two, and three WT units with OPF using the mPDO algorithm reduces the total energy loss over 24 h to 679.6316 kWh, 560.7521 kWh, and 539.2907 kWh, respectively, as listed in Table 5. The net energy injected into the grid by installing one, two, and three WT units amounts to 19,814.4213 kWh, 24,396.5031 kWh, and 27,469.4198 kWh, respectively. Unlike PV systems, the integration of three WT units achieves a significant reduction in daily active power loss, with a PLMI of 75.1936, as depicted in Fig. 16. This is attributed to the fact that WT units can supply apparent power (active and reactive), whereas PV systems only supply active power to the utility. The penetration level of renewable PV and WT is also noteworthy, with the highest penetration observed in the case of WT, particularly when three WTs are installed, reaching 51.4857%.

**Table 5:** Optimal incorporation of single and multiple PV and WT units integrated into 69-bus under commercial load type using mPDO algorithm.

	Bus/size/OPF	Capacity	Total PV	Total energy	PL	Energy loss	PLMI
	kW	kWh	kW	kWh	%	kWh	%
Without DG	–	–	–	–	–	2173.9944	–
1-PV	61/2168.2381/1	16,319.1394	2168.2381	16,319.1394	30.5868	1124.5492	48.2727
2-PVs	17/611.5703/1	4602.9544	2674.6247	20,130.4336	37.7303	1038.2363	52.2429
	61/2063.0544/1	15,527.4792					
3-PVs	11/599.5049/1	4512.1443	3030.4973	22,808.8910	42.7505	1021.7016	53.0035
	17/439.5344/1	3308.1343					
	61/1991.458/1	14,988.6124					
1-WT	61/2097.3155/0.8158	19,814.4213	–	19,814.4213	37.1380	679.6316	68.7381
2-WTs	17/589.7223/ 0.8287	5571.4105	–	24,396.5031	45.7261	560.7521	74.2064
	61/1992.5971/0.8149	18,825.0926					
3-WTs	11/553.0996/0.81611	5225.4176	–	27,469.4198	51.4857	539.2907	75.1936
	17/429.4170/0.8325	4056.9241					
	61/1925.0646/0.8148	18,187.0781

**Figure 17:**
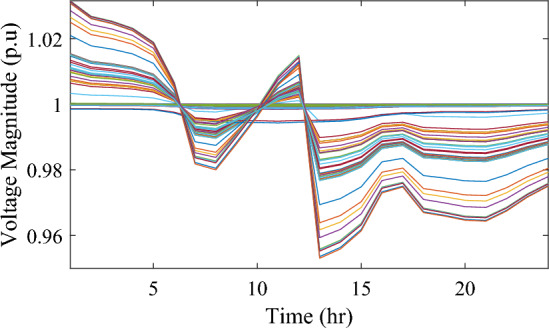
Voltage profile of 69-bus DN during integrating three WTs for the commercial load type.

Figure 17 illustrates the voltage levels of the 69-bus system when three WTs are deployed at OPF. The inclusion of three WTs helps maintain the bus voltages within acceptable limits. Notably, there is a significant improvement in voltage levels when WT power is available, while a slight decline occurs during periods when WT power is absent, especially from 6 p.m. until the end of the day. In conclusion, the optimal deploying non-dispatchable renewable WT and PV systems into the 69-bus test network, accounting for their uncertainties and utilizing the time-varying commercial load model, significantly enhances loss reduction and voltage profile, highlighting the effectiveness of the mPDO algorithm.

#### Case #3: stochastic optimal planning of PV and WT under residential demand

**Figure 18:**
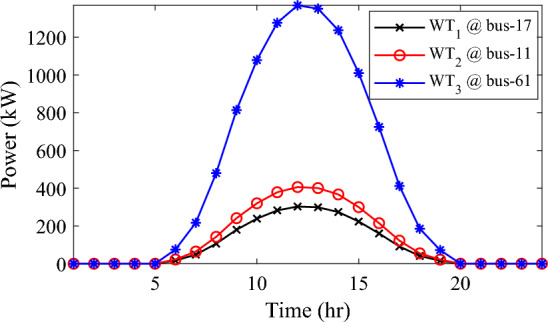
PV output power at nodes11, 17 and 61 using the mPDO algorithm during installing three PVs for the residential load.

**Figure 19:**
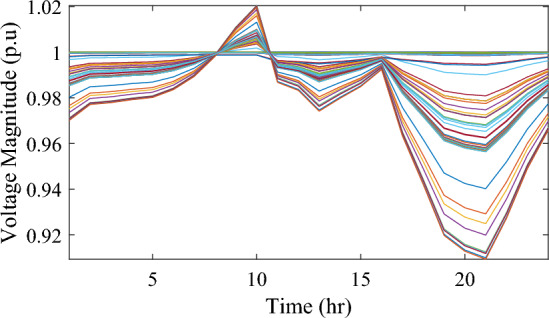
Voltage profile of 69-bus DN during integrating three PVs for the residential load type.

In this case, the summation of the reactive and active loads over 24 h amount to 34,428.4221 kVAr and 48,570.9576 kW, respectively. Similarly, without the integration of WT or PV units into the test system, the total apparent power loss for 24 h is 1868.0756 kW + 850.4165 kVAr. The results presented in Table 6 demonstrate that incorporating a single PV unit at node 61 alleviates the active power loss of the network by 25.6221%, resulting in a daily loss of 1389.4353 kWh. Moreover, by strategically locating and sizing two PVs at buses 17 and 61, the system loss decreases to 1349.2406 kW, representing an improvement of approximately 2% compared to the integration of a single PV unit. Furthermore, deploying three PVs at nodes 11, 17, and 61 with a net power of 2,077.9086 kW decreases the active power loss by 28.1783% compared to the integration of two PVs. The energy generated by a single, two, and three PVs in the test network is 11,207.1613 kWh, 13,823.8395 kWh, and 15,639.2792 kWh, respectively. Figure 18 visualizes the solar PV-generated power during the day when three units are located at nodes 11, 17, and 61. However, as described in Fig. 19, the voltage profiles of the test network violate the minimum acceptable limits, indicating that even the best incorporation of three PV units cannot meet the strictest voltage requirements in this load profile. For example, node 65 experiences a voltage level as low as 0.9128 p.u. at 8 p.m. and 0.9093 p.u. at 9 p.m.

**Table 6:** Optimal incorporation of single and multiple PV and WT units integrated into 69-bus under residential load type using mPDO algorithm.

	Bus/size/OPF	Capacity	Total PV	Total energy	PL	Energy loss	PLMI
	kW	kWh	kW	kWh	%	kWh	%
Without DG	–	–	–	–	–	1868.0756	–
1-PV	61/1489.0365/1	11,207.1613	1489.0365	11,207.1613	23.0738	1389.4353	25.6221
2-PVs	17/419.2299/1	3155.3135	1836.7008	13,823.8395	28.4611	1349.2406	27.7738
	61/1417.4709/1	10,668.526					
3-PVs	11/405.9945/1	3055.6983	2077.9086	15,639.2792	32.1988	1341.6839	28.1783
	17/302.9161/1	2279.8836					
	61/1368.998/1	10,303.6973					
1-WT	61/1316.8861/0.8164	12,441.3026	–	12,441.3026	25.6147	1313.3289	29.6962
2-WTs	17/365.5528/0.8290	3453.5655	–	15,292.6953	31.4853	1268.3281	32.1051
	61/1253.1474/0.8156	11,839.1298					
3-WTs	11/338.2781/0.81515	3195.8881	–	17,179.1904	35.3693	1260.2829	32.5358
	17/268.2247/0.8339	2534.0573					
	61/1211.8789/0.8156	11,449.245

**Figure 20:**
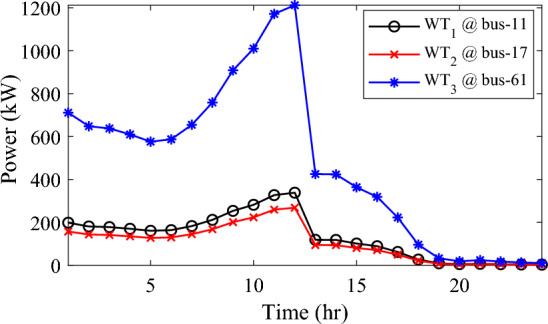
WT output power at nodes11, 17 and 61 using the mPDO algorithm during installing three WTs for the residential load.

**Figure 21:**
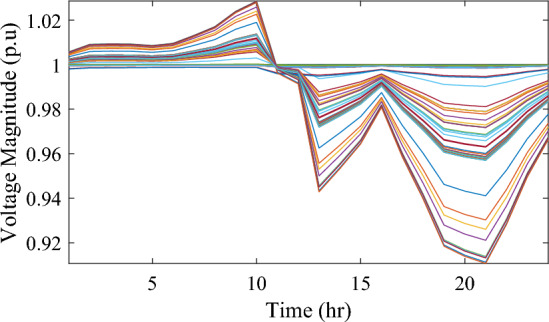
Voltage profile of 69-bus DN during integrating three WTs for the residential load type.

The integration of WTs yields a significantly better reduction in energy loss, as evidenced in Table 6. Using the mPDO optimizer, the total active power loss for one day is mitigated to 1313.3289 kWh, 1268.3281 kWh, and 1260.2829 kWh by incorporating one, two, and three WT units, respectively, into the 69-bus network. The injected energies from the WT units are 12,441.3026 kWh, 15,292.6953 kWh, and 17,179.1904 kWh for one, two, and three units, respectively. Figure 20 illustrates the optimized WT profiles located at buses 11, 17, and 61, each with an OPF of 0.81515, 0.8339, and 0.8156, respectively. The PL of WT unit is 25.6147%, 31.4853%, and 35.3693% for one, two, and three units, respectively. The voltage magnitudes of the test network, shown in Fig. 21, indicates that the voltage magnitudes in most nodes remain within 5% of the nominal value, particularly after 7 p.m., when WT power generation is absent. Consequently, the integration of non-dispatchable renewable WT and PV systems, considering their uncertainties with the time-varying load model, leads to slight improvements in loss reduction and voltage profile within the 69-bus test network.

#### Case #4: stochastic optimal planning of PV and WT under industrial demand

In the fourth scenario, the mPDO algorithm is adopted to address the scheduling of single and multiple PVs and WTs, considering the uncertainty of the industrial load model in the 69-bus, as well as the intermittence of solar irradiance and wind speed. This load profile exhibits a daily active demand of 50,285.8976 kW and a reactive demand of 35,644.0184 kVAr. With the original case, the total active power loss over a practical day is 1,890.2107 kWh, and the reactive power loss is 860.6678 kVAr.

**Figure 22:**
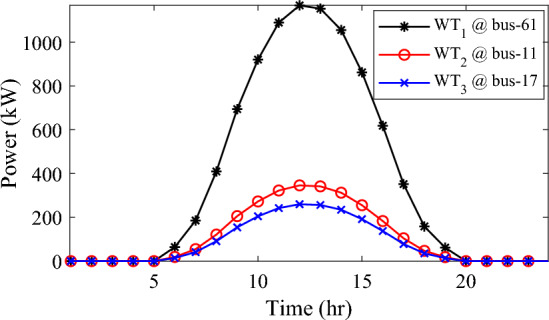
PV output power at nodes11, 17 and 61 using the mPDO algorithm during installing three PVs for the industrial load type.

Similar to the previous scenario, the mPDO is employed to identify the optimal inclusion of PVs and WTs in order to minimize energy loss. According to the results presented in Table 7, incorporating a single PV unit at node 61 reduces the energy loss of the network to 1553.5745 kWh, resulting in a reduction of 17.8095%. By strategically placing two PVs at buses 17 and 61, the energy loss is further reduced to 1524.4738 kWh, achieving a slight improvement of approximately 1.6% compared to a single PV unit. Additionally, deploying three PVs with a total capacity of 1,773.5428 kW at nodes 11, 17, and 61 (depicted in Fig. 22) leads to a reduction in energy loss of 19.6372%.

**Table 7:** Optimal incorporation of single and multiple PV and WT units integrated into 69-bus under industrial load type using mPDO algorithm.

	Bus/size/OPF	Capacity	Total PV	Total energy	PL	Energy loss	PLMI
	kW	kWh	kW	kWh	%	kWh	%
Without DG	–	–	–	–	–	1890.2107	–
1-PV	61/1270.6715/1	9563.6475	1270.6715	9563.6475	19.0185	1553.5745	17.8095
2-PVs	17/358.8492/1	2700.8613	1568.3033	11,803.7588	23.4733	1524.4738	19.349
	61/1209.4541/1	9102.8975					
3-PVs	11/345.8366/1	2602.9223	1773.5428	13,348.4840	26.5452	1519.0259	19.6372
	17/259.7370/1	1954.8982					
	61/1167.9692/1	8790.6635					
1-WT	61/1271.7867/0.8156	12,015.2254	–	12,015.2254	23.8938	1374.7980	27.2675
2-WTs	17/357.8584/0.82846	3380.8726	–	14,807.2747	29.4462	1331.6519	29.5501
	61/1209.461/0.81477	11,426.4021					
3-WTs	11/336.1548/0.81586	3175.8282	–	16,677.8832	33.1661	1323.7414	29.9686
	17/260.8481/0.8326	2464.3663					
	61/1168.3165/0.8146	11,037.6887

**Figure 23:**
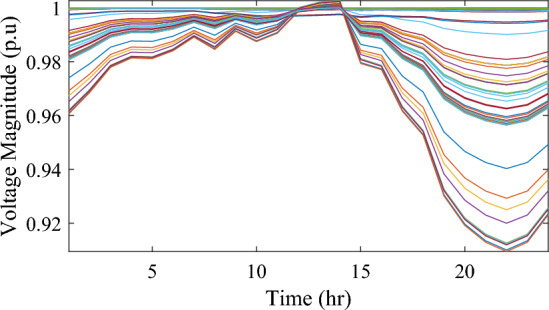
Voltage profile of 69-bus DN during integrating three PVs for the industrial load type.

The total active power output over 24 h for the PV system is 9,563.6475 kWh, 11,803.7588 kWh, and 13,348.4840 kWh, respectively, for the incorporation of one, two, and three PVs into the 69-bus DN. The voltage profile of the present test system after including multiple PVs using the mPDO is illustrated in Fig. 23. As seen in this figure, the optimum distribution of three PVs does not completely meet the voltage restrictions, specifically after 6 p.m.

**Figure 24:**
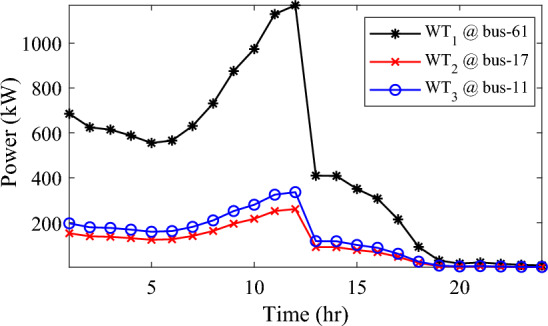
WT output power at nodes11, 17 and 61 using the mPDO algorithm during installing three WTs for the industrial load type.

**Figure 25:**
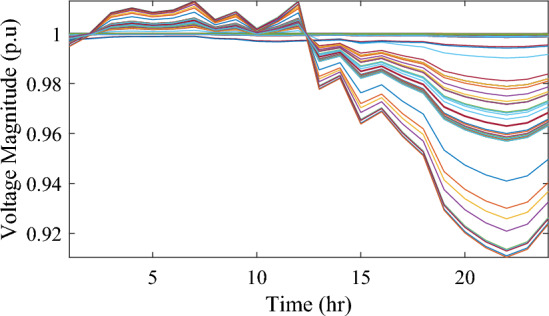
Voltage profile of 69-bus DN during integrating three WTs for the industrial load type.

As shown in Table 7, the mPDO is also applied to optimize the distribution of single and multiple WTs with the OPF in the 69-bus system under the industrial load pattern. The integration of 1, 2, or 3 WT units in the network results in a reduction of the daily active power loss by 1,374.7980 kWh, 1,331.6519 kWh, and 1,323.7414 kWh, respectively, with corresponding percentage reductions of 27.2675, 29.5501, and 29.9686. Furthermore, the injected energies of 12,015.2254 kWh, 14,807.2747 kWh, and 16,677.8832 kWh are achieved by incorporating one, two, and three WT units, respectively. The WT profiles are depicted in Fig. 24, with an optimal distribution at bus 11, bus 17, and bus 61, along with OPF of 0.81586, 0.8326, and 0.8146, respectively. Figure 25 illustrates the voltage profile of the test system, demonstrating that after 7 p.m., most nodes have voltage magnitudes lower than 5% of the nominal value due to the limited WT power production. Therefore, the optimum planning of WT and PV systems, considering the time-varying industrial demand model, leads to a partial enhancement in the loss alleviation and bus voltage magnitude of the 69-bus test network.

#### Case #5: stochastic optimal planning of PV and WT under mixed demand

**Figure 26:**
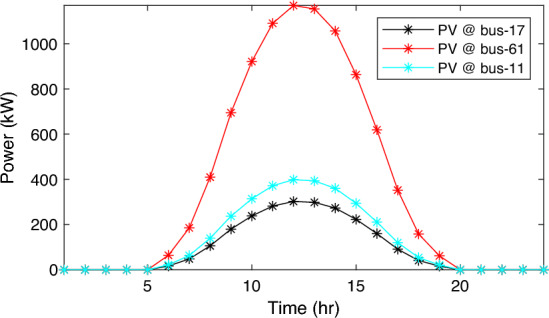
PV output power at nodes11, 18 and 61 using the mPDO algorithm during installing three WTs for the mixed load type.

**Figure 27:**
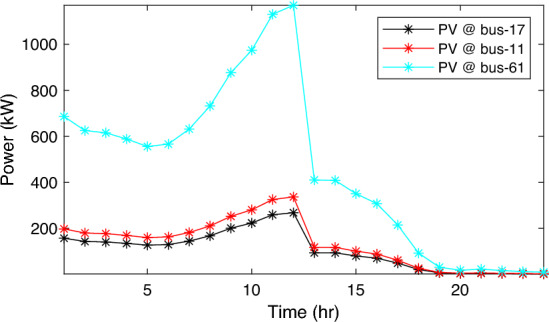
WT output power at nodes11, 17 and 61 using the mPDO algorithm during installing three WTs for the mixed load type.

In this case study, the optimized 69-bus DN encloses a mix of residential (buses 1–27, buses 47–52, buses 66–69), industrial (buses 26–33), and commercial loads. At the base scenario, the total kWh and kVAr are 49,694.0853 and 35,226.1325, respectively. The developed algorithm is adopted to offer the optimal inclusion of three PVs and WTs into the present system under a mixed load profile, as listed in Table 8. The best integration of three PVs decreases the energy loss to 1474.5365 kWh with a PLMI and Pl of 21% and 28.3148, respectively. On the other hand, the optimal deployment of three WTs operated with OPF mitigates the daily active power loss to 1297.7713 kWh with an enhancement of PLMI of 9.5% and an increased PL of 5.5% against the PV system. Further, the curve of the generated output power of the PV and WT installed at buses 11, 17, and 61 are displayed in Figs. 26 and 27, respectively.

**Table 8:** Optimal incorporation of single and multiple PV and WT units integrated into 69-bus under mixed load type using mPDO algorithm.

	Bus/ Size /OPF	Capacity	Total PV	Total Energy	PL	Energy Loss	PLMI
	kW	kWh	kW	kWh	%	kWh	%
Without DG	–	–	–	–	–	1867.9163	–
3-PVs	11/398.4841/1	2999.1712	1869.5111	14,070.7853	28.3148	1474.5365	21.0598
	17/302.1695/1	2274.2644					
	61/1168.8575/1	8797.3497					
3-WTs	11/336.8435/0.81112	3182.3348	–	16,763.032	33.7324	1297.7713	30.523
	17/268.1479/0.83557	2533.3311					
	61/1169.3409/0.8148	11,047.3661

**Figure 28:**
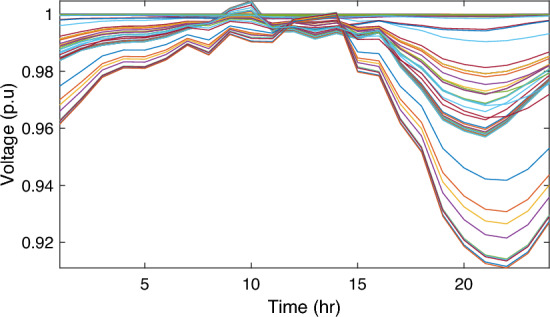
Voltage profile of 69-bus DN during integrating three PVs for the mixed load type.

**Figure 29:**
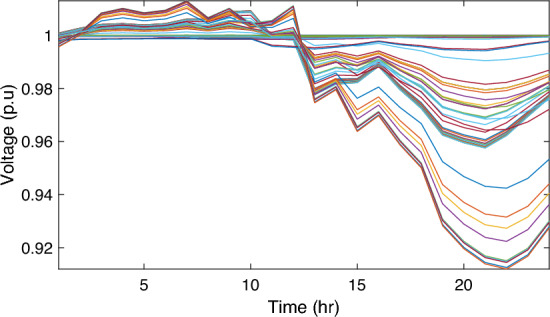
Voltage profile of 69-bus DN during integrating three WTs for the mixed load type.

Figures 28 and 29 display the voltage profile during one practical day after incorporating multiple PVs and WTs for the mixed load demand. Because output power is frequently only available between 6:00 a.m. and 7:00 p.m. for PV systems and between 12:00 a.m. and 6:00 p.m. for WT, the system's voltage profile is confined under the minimal limit, especially from 7:00 p.m. to 12:00 a.m. As a result, the type of load and DG technology has a significant influence on the voltage profile of the distribution grids.

#### Case #6: synchronous optimum PV and WT allocation for the different load models

**Figure 30:**
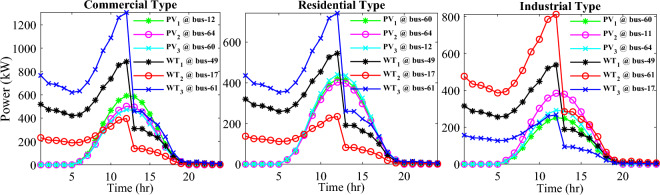
WT and PV output power curves using the mPDO algorithm during simultaneous integration of three WTs and three PVs for different load type.

Unlike the previous scenarios, the mPDO is applied to study the impacts of different dynamic load profiles on the performance of the 69-bus DN, considering the simultaneous plaining of multiple PVs and WTs (as listed in Table 9). Figure 30 illustrates the power output curves of the integrated PVs and WTs for three different load patterns: commercial, residential, and industrial.

**Table 9:** Simultaneous integration of multiple PV and WT units into 69-bus DN using the mPDO algorithm under uncertainty for different load type.

	DG allocation			Unit capacity	Total capacity	Total energy	PL	Energy loss	PLMI
	Bus	Capacity	OPF	kWh	kW	kWh	%	kWh	%
Commercial type	12	592.1657	1	4456.9068	1566.6621	11,791.4062	67.9059	395.9191	81.7884
	60	472.8394	1	3558.8030					
	64	501.6569	1	3775.6964					
	17	396.6760	0.7000	3747.6024	-	24,438.7642			
	49	884.2427	0.8125	8353.8972					
	61	1305.8739	0.7000	12,337.2647					
Residential type	12	441.0704	1	3319.6948	1264.1240	9514.3681	49.1827	1147.4072	38.5781
	60	419.9252	1	3160.5468					
	64	403.1284	1	3034.1265					
	17	234.3979	0.7000	2214.4776	-	14,374.1623			
	49	544.6564	0.8115	5145.6495					
	61	742.4211	0.7000	7014.0352					
Industrial type	11	384.3114	1	2892.5010	928.1752	6985.8657	44.2924	1279.5147	32.3084
	60	251.6425	1	1893.9754					
	64	292.2213	1	2199.3893					
	17	268.1524	0.7373	2533.3738	-	15,287.0024			
	49	537.8695	0.8126	5081.5304					
	61	812.0757	0.7000	7672.0982

For the commercial load type, three PVs with a net capacity of 1566.6621 kW are installed at nodes 12, 60, and 64. Three WTs are located at bus 17, bus 49, and bus 61, with OPF of 0.7000, 0.8125, and 0.7000 respectively. The total energy generated by the multiple PVs and WTs is 11,791.4062 kWh and 24,438.7642 kWh, respectively, resulting in a PL of 67.9059%. By deploying multiple PVs and WTs simultaneously, a substantial reduction in losses is accomplished under the time-varying commercial load pattern. The loss reduction amounts to 395.9191 kWh, which corresponds to 81.7884% improvement.

Using the mPDO algorithm, the appropriate sizing and placement of three PVs and three WTs are optimized to address the residential and industrial load requirements. This optimization results in energy loss reductions of 1147.4072 kWh and 1279.5147 kWh, with PLs of 49.1827% and 44.2924% respectively.

**Figure 31:**
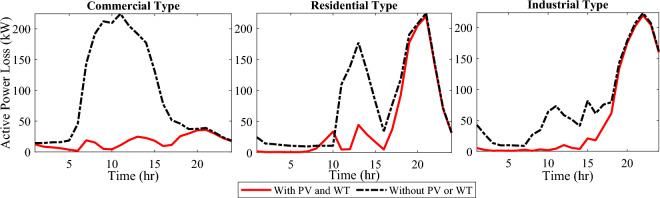
Daily active power loss for different loads of the 69-bus with and without simultaneous integration of three WTs and three PVs using the mPDO algorithm.

**Figure 32:**
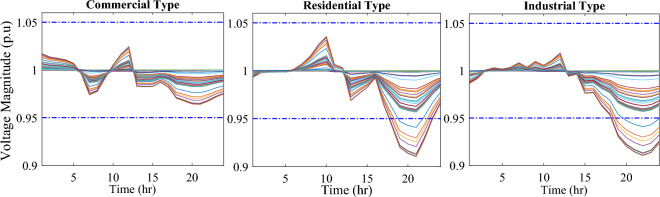
Voltage profile of 69-bus DN during simultaneous integration of three WTs and three PVs for different load type.

Figures 31 and 32 depict the daily active power loss and system voltage profiles for different load patterns in the 69-bus system when multiple WTs and PVs are integrated simultaneously. These figures show that the integration of PVs with WTs improves the voltage profile and reduces energy loss to a lesser extent for the residential and industrial load profiles, in comparison to the commercial load profile. It can be observed from Fig. 31 that the performance of the distribution system is highly influenced by the dynamic load behaviour and the type of DG technology. For instance, the optimal inclusion of multiple WTs and PVs simultaneously satisfies the voltage requirements for the commercial load profile. However, the voltage levels drop below the allowable voltage range for residential and commercial loads for more than three hours during the day.

**Figure 33:**
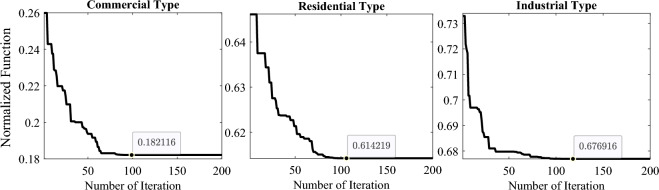
Convergence characteristics of the mPDO algorithm for simultaneous integration of three WTs and three PVs for different load type.

In addition, Fig. 33 illustrates the convergence curves of the mPDO algorithm for the three different load profiles. This provides further evidence that the mPDO method is capable of solving the optimum allocation of DGs under both deterministic and probabilistic situations with a high degree of efficiency and superiority.

## Conclusion and future work

This paper has studied the significant impacts of various dynamic load behaviors on the performance of the distribution system and the planning of the PV-based and WT-based DGs. This has been accomplished using a modified prairie dog optimization algorithm that incorporates a new exploration phase inspired by the slim mold algorithm. Efficient probability distribution models are expressed to model the uncertainty associated with solar irradiation and wind velocity. The optimization model considers practical requirements to minimize energy loss in the distribution network. The effectiveness of the mPDO algorithm has been verified with the aid of the cec2020 benchmark functions, and a comparative study with the original PDO and other recognized algorithms is accomplished through multiple statistical metrics. The deterministic optimal inclusion of single and multiple PV and WT in the 69-bus DN is specified to mitigate active power loss. The simultaneous deployment of PVs and WTs in the test system is also studied, considering commercial, residential, industrial, and mixed load profiles. The simulation results demonstrated that generation and demand uncertainty had a significant impact on distribution grid performance and optimal DG inclusion. The developed algorithm successfully achieves high penetration levels for synchronous integration of WT and PV units under different load demands, leading to significant energy savings. As an illustration, the penetration levels for industrial, residential, and commercial loads are 44.2924%, 49.1827%, and 67.9059%, respectively, with PLMI of 32.3084%, 38.5781%, and 81.7884%.

The proposed optimizer could be applied to address various complex optimization problems in future research. Furthermore, the integration of dispatchable sources, such as biomass, with wind turbine generators will be explored to enhance their dispatchability and overall system reliability. Additionally, potential future work includes incorporating energy storage systems, utilizing dynamic load models, exploring multi-objective optimization approaches, conducting comprehensive economic analyses, and assessing the impact of renewable energy integration on grid resilience and stability.

Future work on the mPDO can explore several promising directions to enhance its capabilities and expand its applicability. One potential avenue is the development of a binary version of the mPDO, which would enable it to handle discrete optimization problems such as feature selection, clustering, and the knapsack problem more effectively. Additionally, creating a multi-objective version of the mPDO can facilitate its application to problems with multiple conflicting objectives, allowing for a more comprehensive optimization process in complex scenarios.

Furthermore, the incorporation of advanced operators, such as local escape operators and opposition-based techniques, can be investigated to improve the optimizer's ability to balance exploration and exploitation. These enhancements could help the mPDO avoid local minima and better navigate the solution space, leading to more robust optimization performance. By pursuing these improvements, the mPDO can be made more versatile and powerful, opening up new possibilities for its application across a broader range of optimization problems.

## Data Availability

The datasets generated during and/or analyzed during the current study are available from the corresponding author on reasonable request.

## Abbreviations

PV: Photovoltaic
WT: Wind turbine
DG: Distributed generator
UPF: Unity power factor
OPF: Optimal power flow
TVD: Total voltage deviation
SVSI: Summation voltage stability index
PDO: Prairie dog optimization
SMO: Slim mould optimization
BetaPDF: Beta probability distribution function
std: Standard deviation
WeibullPDF: Weibull probability distribution function
APLS: Active power loss sensitivity
PLMI: Power loss mitigation index
PL: Penetration level
DN: Distribution network
BES: Battery energy storage system
